# Crustal Strain Patterns Associated With Normal, Drought, and Heavy Precipitation Years in California

**DOI:** 10.1029/2020JB019560

**Published:** 2021-01-04

**Authors:** Jeonghyeop Kim, Alireza Bahadori, William E. Holt

**Affiliations:** ^1^ Department of Geosciences Stony Brook University Stony Brook NY USA

## Abstract

We invert continuously operating Global Positioning System (cGPS) data obtained between 2007 and 2019 to quantify non steady‐state horizontal strain anomalies in California. Our long‐wavelength transient strain model shows seasonal and multiannual variations in horizontal strain anomalies within the plate boundary zone. During the summer, in general, a zone of extensional dilatation develops along the San Andreas Fault zone and Sierra Nevada, whereas contractional dilatation develops along the Eastern California Shear Zone (ECSZ) north of 36.5°N. The patterns of dilatational strain are opposite during the winter. We find that these seasonal strain anomaly patterns vary in magnitude, depending on precipitation intensity in California. Investigating hydrologic loading models and their horizontal elastic responses reveal that water mass loads on the surface from the precipitation in California are the major sources of the observed long‐wavelength horizontal transient strains. We show, however, that a heavy damping in the inversion of the cGPS data is required for the long‐wavelength horizontal strain solutions to best match with the expected elastic response from hydrologic loading. Appropriate fitting of the horizontal cGPS yields amplified horizontal strain signals in the Sierra Nevada, along regions adjacent to the San Andreas Fault, and within the ECSZ. The larger‐than‐expected amplitudes may be associated with poroelastic responses or thermoelastic changes that are superimposed on the hydrologic response. We demonstrate that there is a persistent sharp boundary of horizontal dilatational strain domains at the transition between the High Sierra and Basin and Range Province, caused by the sharp gradient in hydrologic loading there.

## Key Points

- We invert cGPS data to quantify seasonal horizontal strain field in California for normal, drought, and heavy precipitation years
- The long‐wavelength transient strain inferred from cGPS can be explained by the Earth's elastic responses to hydrologic loading
- There exist localized, high‐amplitude strain anomalies, superimposed on the long‐wavelength field, repeating reliably over years

## 1 Introduction

Continuously operating Global Positioning System (cGPS) measurements have detected seasonally driven transient deformation within the crust, including the Earth's elastic response to hydrologic loads, temperature gradients, and atmospheric pressure (e.g., Argus et al., 2014; Ben‐Zion & Allam, 2013; Fu & Freymueller, 2012; Fu et al., 2013; Heki, 2001; Tregoning et al., 2009; van Dam et al., 2001; van Dam and Wahr, 1987; Wahr et al., 2013). Anthropogenic impacts on the surface load, such as groundwater pumping (e.g., Amos et al., 2014), can also cause seasonal transient deformations. Furthermore, seasonal loading patterns may influence seismicity rates within plate boundary zones worldwide (e.g., Bettinelli et al., 2008; Christensen et al., 2007; Gao et al., 2000; Gonzalez et al., 2012; Heki, 2003; Johnson et al., 2020; Luttrell et al., 2007).

Using the dense coverage of Network of the Americas (NOTA) of cGPS (previously Plate Boundary Observatory [PBO]) and other networks, a number of studies have used vertical component time series to investigate the influence of hydrologic loading within the plate boundary zone region in California (Amos et al., 2014; Argus et al., 2014, 2017; Borsa et al., 2014; Johnson et al., 2017a, 2017b). The seasonal peak‐to‐peak amplitudes of vertical movements observed in California can be explained by the solid Earth's elastic response (Farrell, 1972) to the surface load from the precipitation patterns (Argus et al., 2014). California typically has wet winters. Thus, during the winter total water storage on the surface is generally at a maximum level, and this extra load leads to the lowest vertical heights of the elastic crust. During the summer, the crust in California typically reaches the highest vertical point due to the loss of surface loads during the dryer months (Argus et al., 2014). Using vertical cGPS data, Johnson et al. (2017a) showed that the seismicity rates in northern California are modulated by the seasonal stress changes. Using horizontal cGPS data, Kreemer and Zaliapin (2018) quantified seasonal horizontal transient deformation patterns in California and investigated links between the associated transient stress changes and seismicity. Kreemer and Zaliapin (2018) and Johnson et al. (2017a) calculated the monthly averaged signal using a time series spanning several years. However, seasonal hydrologic loading varies from year‐to‐year and prolonged drought also impacts the transient nontectonic deformation field (Argus et al., 2017; Borsa et al., 2014). For example, these studies reported multiannual uplift of the crust during the severe drought between 2012 and 2015 in California. Using vertical and horizontal cGPS data, Hammond et al. (2019) investigated the implications of drought effects for seismicity rate and magmatic inflation in the Long Valley Caldera area. Given the large observed changes in multiannual signals inferred from primarily vertical component cGPS, it is important to quantify how such multiannual variations in hydrologic loading influence the horizontal strain field in the crust throughout the entire plate boundary zone. Such a quantification of time‐dependent horizontal strain changes will be important for advancing our understanding of time‐variable stress changes and their potential influence on seismicity.

We invert horizontal cGPS data of NOTA to quantify a 13‐year history of horizontal transient strain patterns between 2007 and 2019 within the plate boundary zone in California. Our solutions involve horizontal strain, not strain rates, as we only analyze displacements. Although the vertical component of cGPS data is not directly used for the horizontal transient strain model, we employ it to identify and eliminate stations affected by nonelastic sources (see Section 2.1). Here, we present two different solutions: one is a long‐wavelength strain solution that requires heavy damping in our inversion of cGPS data, and the other is a shorter‐wavelength, higher‐amplitude solution that we obtained by fitting the cGPS data closely. Our region of analysis includes most of the Great Valley, the Sierra Nevada, the Eastern California Shear Zone (ECSZ), and the San Andreas Fault zone (SAF) south of latitude 39°N.

Our first (long‐wavelength) solution is statistically significant for the entire duration of our analysis between January 2007 and June 2019. This solution highlights remarkable seasonal strain anomaly patterns and their multiannual variations in magnitude during drought and heavy precipitation years. We investigate the link between the history of the long‐wavelength horizontal strain anomalies and the solid Earth's elastic response to various hydrologic loading patterns, comparing our results with two different hydrologic loading models. Our analysis reveals that loading‐driven seasonal horizontal strain changes can vary substantially in space and time from year‐to‐year, depending on patterns and magnitude of precipitation. Our model resolves notable drought effects on deformation patterns between 2012 and 2015, as well as the impact of heavy precipitation in years of 2011, 2017, and 2019 on the regional patterns of transient crustal strain.

However, the heavy damping used in our inversion of horizontal cGPS data, to obtain the long‐wavelength solution, smooths out and masks physically meaningful local features in the horizontal transient strain field. To capture the full seasonal horizontal field observed in the cGPS data, we produce the second solution (shorter‐wavelength, higher‐amplitude). Although the statistical significance of the individual monthly solutions is lower than the long‐wavelength solution, the shorter‐wavelength, higher‐amplitude seasonal strain patterns generally repeat throughout the 13‐year history. Thus, we stack the solutions over years and present a reliable shorter‐wavelength, higher‐amplitude component of the seasonal strain anomaly patterns. These seasonal transient strain patterns are superimposed on the long‐wavelength patterns, showing more pronounced local features across the entire region of the analysis.

## 2 Methods

### 2.1 Data

We analyze NOTA cGPS observations in California, which are operated and archived by the NSF's GAGE Facility at UNAVCO. We use NOTA position time series products processed by the Nevada Geodetic Laboratory (NGL) (Blewitt et al., 2018), which are available through the entire time of our research. We also analyze another level‐2 position time series data processed by the GAGE Facility (Central Washington University [CWU]) (Herring et al., 2019). In addition to the solution presented in the main text (derived from NGL products), we provide another solution (derived from CWU products) in the supporting information (Figures S1–S3). There are differences between how the NGL and GAGE facilities process and determine reference frame for the generation of their products. These differences between the solutions can lead to potential biases (Herring et al., 2016). For instance, NGL removes a scale from their product, which suppresses the height signal. The GAGE solution does not suppress the height signal, which leads to a larger vertical seasonal signal compared to NGL. GAGE and NGL solutions also use slightly different reference frames for rotation from the IGS08 (Rebischung et al., 2012) to North America frame (Altamimi et al., 2012; Blewitt et al., 2013; Herring et al., 2016). Owing to these differences, a best approach is to treat these solutions from the different processing centers separately. Although we find minor differences in crustal strain solutions using data from these separate analysis centers, patterns of deformation are generally consistent spatially and temporally.

**Figure 1:**
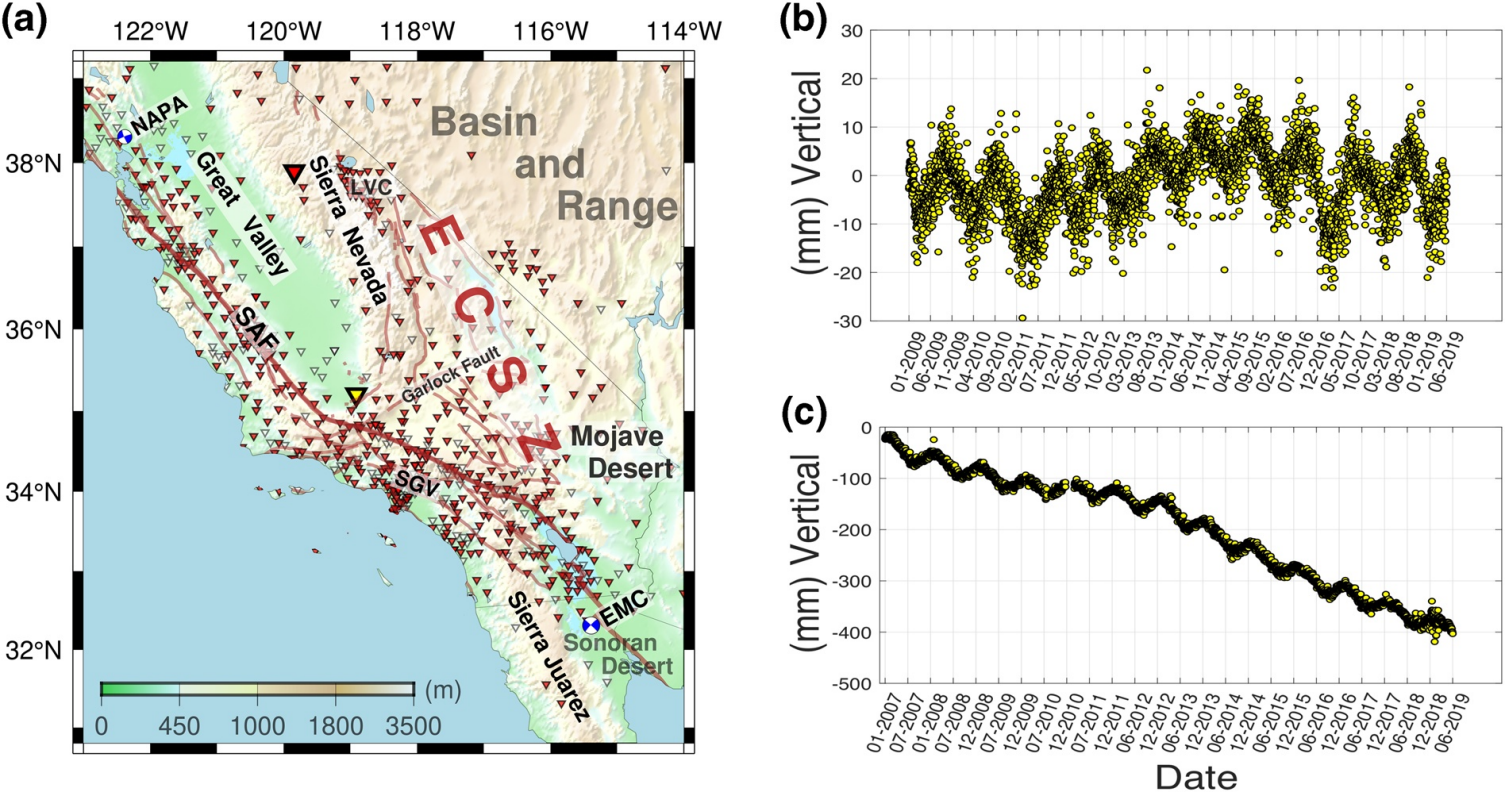
(a) A map of the locations of cGPS stations. The red inverted triangles represent the stations we used. The yellow inverted triangles indicate eliminated stations based on the analysis of vertical displacement time series (Argus et al., 2014). We remove all the stations that show maximum heights in months between November and April and the stations that have subsidence rates less than −1 mm/year between 2012 and 2015. (b) The vertical time series of station P308, represented as an example of the cGPS stations that show the Earth's elastic response to the loading. Note that the time series show local minimums of height every winter or early spring (from March to April) as well as multiannual uplift motion during the severe drought between 2012 and 2015. (c) Vertical time series from station ARM2, which demonstrates an example for stations that are suspected of being affected by poroelastic processes. The local maximums of height appear every winter or early spring. A significant subsidence lasts during the entire time, and is exacerbated during the drought. The locations of the two stations P308 (https://doi.org/10.7283/T5R20ZCV) (UNAVCO Community, 2008) and ARM2 (https://doi.org/10.7283/T5D798PN) (Hudnut et al., 2006) are shown in panel (a) as the largest red and yellow inverted triangles, respectively. The focal mechanisms in panel (a) are for the 2010 El Mayor‐Cucapah Earthquake (EMC) and the 2014 South Napa Earthquake (NAPA) from Global Centroid‐Moment‐Tensor project (Dziewonski et al., 1981; Ekström et al., 2012). The background is from the Global relief model ETOPO 1 (Amante & Eakins, 2009). ECSZ, Eastern California Shear Zone; LVC, Long Valley Caldera; SAF: San Andreas Fault; SGV, San Gabriel Valley.

We use horizontal cGPS positions from the total of 678 stations to evaluate and estimate the potential horizontal transient strains in California. We initially investigate 908 cGPS stations between 2007 and 2019. We conservatively identify and eliminate 230 stations that show evidence for poroelastic responses (Figure 1). To identify such stations, we use vertical components of displacement time series. We first take monthly averaged vertical time series values for each station and carefully determine the timing of maximum averaged height and peak‐to‐peak amplitude over 12 years. We eliminate all the stations that experience maximum uplifts between November and April, which indicate that the responses are mainly affected by poroelastic influences, as opposed to elastic loading effects (Argus et al., 2014). Moreover, to distinguish the stations affected by groundwater pumping, we calculate the average subsidence rates over the severe drought years between 2012 and 2015 (Argus et al., 2014). If the subsidence rates during this time span are larger than 1 mm/year, we interpret that the stations are affected by anthropogenic activity (Argus et al., 2014) and we omit them (Figure 1c). Argus et al. (2014) used a 3 mm/year cutoff, and thus our cutoff is more conservative.

Using the threshold subsidence rate of 1 mm/year during the severe drought, we identify 63 stations for removal. One possible concern of the 1 mm/year threshold that we use is that stations with a subsidence rate in excess of 1 mm/year may have a background tectonic subsidence (e.g., Hammond et al., 2016) instead of a poroelastic subsidence. Such a station would have an elastic signal, identifiable as a peak seasonal height in summer. We investigated the time series of stations that had subsidence rates between 1 and 3 mm/year and found that 36% had an elastic signal and 64% had no elastic signal. The stations with an elastic signal were situated in regions near the SAF, mostly north of the Bay Area, where the spatial distribution of cGPS is generally dense. These removed stations have little influence on our estimates of the transient nontectonic strain field because there is sufficient coverage from remaining stations (Figure 1).

To compare signals captured by cGPS data with elastic crustal responses to hydrologic loading (e.g., Argus et al., 2014; Borsa et al., 2014), we employ the UNAVCO hydrologic loading position time series (Puskas et al., 2017) derived from the Noah 0.125° grid Land Surface Model for National Land Data Assimilation System (NLDAS; Mitchell et al., 2004). This model is generated using three parameters in the surface model as inputs (soil moisture, snow load, and total vegetation water storage), and thus it is independent from the cGPS observations (Puskas et al., 2017). Their hydrologic model displacements are resolved using Green's function responses (Farrell, 1972; van Dam et al., 2001; Wahr et al., 2013) at each location of the PBO stations (now NOTA) within the surface model grid (Puskas et al., 2017). To further investigate, we also use the surface water estimates of Argus et al. (2017; available at: https://sideshow.jpl.nasa.gov/pub/usrs/argus/water/west.us). These estimates are inferred from vertical cGPS and a composite hydrologic model.

### 2.2 A Moving Time Window of Displacements for Strain Evolution Estimates

It is necessary to convert cGPS position estimates into displacements over a specified time window so that spatial variations in these displacements can be used for transient strain analysis. Kraner et al. (2018) found the 4‐month moving window to be optimal for capturing temporal variations in seasonal strain and we thus adopt the 4‐month moving window. Kraner et al. (2018) found that a moving 6‐month time window did not always capture the peak of seasonal strain change, possibly owing to the fact that the time series are not necessarily sinusoidal but may contain asymmetries.

The 4‐month time‐window is defined as the horizontal displacement at each station between starting and ending time 4 months later, obtained as a difference in monthly averaged position. For example, the first 4‐month interval is between the average for January 2007 and the average for September 2006. The moving window consists of the calculation of strains within 4‐month time‐windows that are successively shifted by 1‐month increments (Kraner et al., 2018). The total number of the 4‐month displacement intervals is 150, starting from January 2007 to June 2019. The interval is labeled by the ending month. For example, the difference in positions over the interval between September 2017 and January 2018 is referred to as the displacements for January 2018. The number of available stations in each time interval varies from 369 to 609 (out of the total pool of 678 stations; Figure S4).

### 2.3 Inversion of Displacements for a Transient Strain Field

We invert the 4‐month displacements using a similar methodology to previous work (Beavan & Haines, 2001; Haines & Holt, 1993; Holt et al., 2000; Holt & Shcherbenko, 2013; Kraner et al., 2018), where the horizontal displacement gradient tensor field (i.e., strains and rotations) is parameterized as a continuous field on the surface of a sphere. The forward model of the continuous field is parameterized using a continuous time‐varying three dimensional (3‐D) rotation vector function W(r,t), where r is a directional cosine vector that passes through the Earth's center, *t* is time, and both W(r,t) and r are perpendicular to the Earth's surface. The continuous horizontal displacement field u(r,t) is

(1) $$u(r,t)=W(r,t)\times r+\omega^{k}\times r,$$

where $\omega^{k}$ is the rotation that translates the *k* ^th^ GPS network into a best‐fit (least‐squares sense) frame, and × denotes the cross product (Beavan & Haines, 2001). For our analyses, we do not solve for the best‐fit frame, but instead we adopt the North America frame provided by the different analysis centers. The number of GPS networks, *k*, is 1 (GAGE or NGL). We use a single inversion (*k* = 1) approach for reasons discussed in Section 2.1 (the two data sets are treated separately in different inversions).

The horizontal spatial derivatives of the displacements in Equation (1) yield model estimates of horizontal strain ${\hat{e}}_{ij}$ on a 0.1° × 0.1° curvilinear grid (Kraner et al., 2018), where the Bessel form of bicubic spline interpolation (deBoor, 1978) is used to define the values and spatial derivatives of W(r,t) at grid nodes (Beavan & Haines, 2001; Haines et al., 1998). The curvilinear grid we use for this study is set up to study transients within the transform‐dominated plate boundary zone in California. The western side of the grid defines a rigid boundary, whereas the interior and eastern boundary cells of the grid are all deformable (Kraner et al., 2018). Our curvilinear grid contains 7,185 deformable cells. Bicubic spline parameters define a continuous W(r,t) throughout the 7,185 deformable cells (Beavan & Haines, 2001).

The values of W(r,t) are determined at the grid knotpoints and throughout the cells in a least squares inversion of observed horizontal strains $\Delta e_{ij}^{\text{obs}}$ and displacements $\Delta u_{i}^{\text{obs}}$. The observed displacements are recorded at cGPS stations, which can be located anywhere within the grid, and not only at the grid knotpoints. Kraner et al. (2018) described a steady‐state tectonic model that consists of a continuous displacement field $\Delta u_{i}^{\text{steady–state}}(x,y)$ and a continuous strain field $\Delta e_{ij}^{\text{steady–state}}(x,y)$ derived from the consensus GPS (UCERF3) observations (Field et al., 2014; Parsons et al., 2013). The observed horizontal strain tensor components within the 7,185 grid cells are cell‐averaged values from $\Delta e_{ij}^{\text{steady–state}}(x,y)$. These “observed” strains, fitted in the inversion, represent a prior model involving the expectation that cGPS motions and the associated strains are from steady‐state tectonics (Figure S5). Thus, in regions where there are no cGPS data, the model resulting from the inversion will give strains $\Delta e_{ij}(x,y,t_{n})$ and displacements $\Delta u_{i}(x,y,t_{n})$ that match with the steady‐state tectonic model for the given time epoch $t_{n}$. Where there are cGPS data that contain transient tectonic or nontectonic motions, then the “observed” strains (steady‐state model) will be misfit in the inversion in order that the model fits the cGPS data. In this case the model estimates of strain, $\Delta e_{ij}(x,y,t_{n})$, will contain transient strain information.

Following the inversion, we remove the steady‐state tectonic model from the continuous field obtained in the inversion. The residual model displacements and strains represent a departure from steady‐state tectonics, owing to seasonal transients, tectonic transients, and errors in the cGPS measurements. In this study, we show that the residual is dominated by seasonal transients. Thus,

(2) $$\begin{array}{l} u_{i_{n}}^{\text{seasonal}}(x,y,t_{n})=\;\Delta u_{i}(x,y,t_{n})-\;\Delta u_{i}^{\text{steady–state}}(x,y),\\ e_{ij_{n}}^{\text{seasonal}}(x,y,t_{n})=\Delta e_{ij}(x,y,t_{n})-\Delta e_{ij}^{\text{steady–state}}(x,y), \end{array}$$

where $u_{i}^{\text{seasonal}}$ and $e_{ij}^{\text{seasonal}}$ are the calculated estimates of non‐steady‐state displacements and strains as a function of space and time; *n* represents the 150 intervals in increments of one month from January 2007 to June 2019; $\Delta u_{i}$and $\Delta e_{ij}$ are the calculated estimates of 4‐month displacements and accumulated strains as a function of space and time; and $\Delta u_{i}^{\text{steady–state}}$ and $\Delta e_{ij}^{\text{steady–state}}$ are the displacement and strain from the steady‐state model (Kraner et al., 2018).

### 2.4 Regularization of the Transient Strain Field Through Damping in the Inversion

In the joint inversion of the strains and the cGPS, as pointed out above, the matching of a displacement field that contains a transient will involve a departure from the prior model (misfit to the steady‐state, prior, strains). The variance‐covariance matrix, assigned to the observed (prior) strains, influences the size of the model misfit (departure) to the observed strains, which may occur when the cGPS contains transient motions. We adjust a single isotropic variance factor embedded within the variance‐covariance matrix of strain to control how much the model can depart from the steady‐state strain to match cGPS (Kraner et al., 2018). In the manuscript we refer to these adjustments of the isotropic variance factor as changes in the damping level. The larger the isotropic variance, the closer the model is able to fit large transient displacements (lower damping). Thus, large variances can lead to a close match to transient GPS motions, and yield a transient strain field that contains high‐amplitude and short‐wavelength anomalies. Because strain variances are large for the prior model in this case of lower damping, the posterior variances for model strain are typically large. A smaller isotropic variance factor (higher damping) can lead to larger misfits to transient GPS motions, and yield a transient strain field that contains smoother and longer‐wavelength anomalies. Because the variances for the prior model are smaller for cases of high damping, the posterior variances for model strain are also typically small.

The inversion problem is regularized through the adjustment in damping level. We quantify data fitting of cGPS using the *a posteriori* standard error of unit weight (SEUW), which is the square root of the reduced *χ* ^2^ statistic, and can be expressed as

(3) $$\mathrm{SEUW}=\sqrt{\frac{\mathrm{SS}}{N_{\mathrm{dof}}}},$$

where SS is the total sum of squares of residuals associated with the cGPS observations and model displacements (see *A.6* in Beavan & Haines, 2001), and *N*_dof_ is the number of degrees of freedom defined as the total number of cGPS observations, counting both the north and east components. Related to the above discussion, larger values of SEUW are associated with higher damping and lead to smoother transient strain solutions.

### 2.5 A Checkerboard Benchmark Test

Using a synthetic checkerboard distribution of dilatational strains (*e*_*xx*_ = *e*_*yy*_; *e*_*xy*_ = 0), we investigate the capability of the NOTA network (the 678 stations) for recovering a known strain field (e.g., Hammond et al., 2016). We add Gaussian errors to the synthetic displacements associated with the checkerboard strain field that are of the same size as the uncertainties in the 4‐month observed cGPS displacements. We invert these synthetic displacements using the same methodology that we apply for the real data in the NOTA network (same grid, same station distribution). Note that the “observed” strains in the inversion contain no information about the checkerboard strains; the synthetic GPS contains displacements only linked to the checkerboard pattern, which represents an unknown transient field that we wish to recover. This test reveals that we can recover the boundaries of a 2° latitude × 4° longitude checkerboard pattern to better than 0.5°, and we capture the amplitude of the anomalies for 44%–74% of the area in California between latitudes of 33°N and 38°N (Figure S6b). We are also able to recover the boundaries of a 2° latitude × 1° longitude checkerboard pattern within 0.5°, where the coverage of stations is dense, but we find the signals smear out in regions with poor data coverage (e.g., The Great Valley; Figure S6d). In a third test, we analyze a checkboard strain field that is the sum of the 2° × 4° and 2° × 1° fields. We view this field as an analogy of the present transient field in California, which contains a shorter‐wavelength strain signal superimposed on a longer‐wavelength strain signal. We apply progressively greater amounts of damping in the inversion of the displacements and investigate the correlation of the model strain fields with the 2° × 4° alone, as well as with the total field, as a function of SEUW (Figure S7d). We achieve a maximum correlation with the 2° × 4°  field with higher damping (SEUW = 3), whereas lower damping (SEUW = 0) provides the exact fit to the synthetic GPS and a maximum correlation with the total (combined) checkerboard strain field (Figure S7d).

### 2.6 Justification of Damping Levels for Two Transient Strain Models From the Inversion

In this study, we present two transient strain field solutions: one that is long‐wavelength (higher damping) and provides close‐to‐optimal agreement with the elastic strain response to hydrologic loading models, and one where we match the data closely (lower damping). In accord with our hypothesis that seasonal transient anomalies are dominated by hydrologic loading, we perform quantitative comparison between model strain fields obtained from our inversion of cGPS (as a function of damping) and predictions associated with the elastic responses to hydrologic loading models. This comparison enables us to confirm that there is a long‐wavelength transient seasonal horizontal field that is likely driven by hydrologic loading. Superimposed on this long‐wavelength field is a shorter‐wavelength, higher‐amplitude seasonal signal with a source that is not as clear as the long‐wavelength signal.

**Figure 2:**
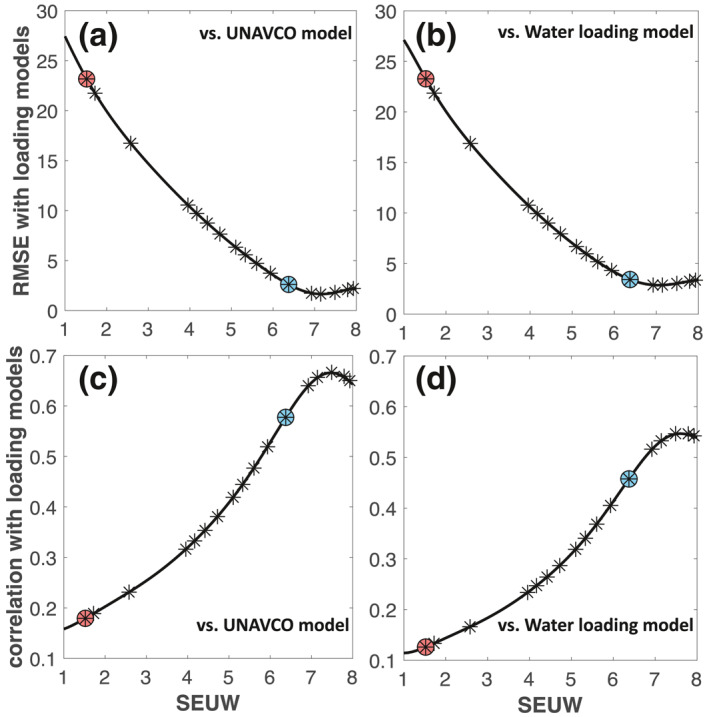
Statistical tests for the comparisons between the strain models from horizontal cGPS and two hydrologic loading models as a function of SEUW. Note that SEUW reflects the misfit to cGPS from the chosen damping level in the inversion (high damping level = high SEUW). Comparison between the models from cGPS and the UNAVCO hydrologic model are presented in the first column panels (a and c); panels (b and d) show the same results between the model from cGPS and the hydrologic model obtained using water loading estimates provided by Argus et al. (2017). Values for RMSE versus SEUW and correlation versus SEUW result from different damping levels in the inversion of cGPS. The 17 asterisks show where the RMSE and correlation values were obtained. The black solid lines are fitting functions to the asterisks using polynomial equations. The blue circled asterisk is the SEUW value of 6.37 that is associated with our chosen damping level for the long‐wavelength transient strain solution presented in Figures 3 and 4. The red circled asterisk indicates the SEUW value of 1.52, which is associated with the damping level for the shorter‐wavelength solution presented in Figures 7c and 7d. cGPS, continuously operating Global Positioning System; RMSE, root mean square error; SEUW, standard error of unit weight.

We calculate the root mean square error (RMSE), and a spatial correlation function, between the models as a function of SEUW (see the Equations 2 and 3 in the supporting information; Figure 2). These comparisons are independent of the slightly different frames of reference between the loading models and the cGPS model in North America frame, since the strains are invariant to reference frames. Maximum correlation and minimum RMSE occur for large damping (SEUW > 7). These results show that a smoothed fit to the horizontal field consistently supports hydrologic loading as a significant source in the long‐wavelength horizontal transient field.

A minimum RMSE between the cGPS‐derived solutions and hydrologic loading models corresponds to an SEUW of 7.14 (Figures 2a and 2b). A high damping level (SEUW of 7.49) gives a maximum in correlation between cGPS‐derived dilatation patterns and hydrologic loading models (Figures 2c and 2d). We choose a damping level that results in an SEUW of 6.37 and an RMSE that is near the minimum. We could choose more substantial damping to achieve even smoother solutions (e.g., SEUW of 7.14), which would result in slightly improved matches to the hydrologic loading models. However, our chosen damping level still captures the long‐wavelength nature of strain signals arising from hydrologic loading models, while it also includes additional signals not present in current hydrologic loading models, but that are well resolved by the network. As an example, we find that the seasonal signals in regions around the SAF are smeared‐out and no longer prominent if we use a damping level that optimizes a fit to the hydrologic loading models.

To capture the full seasonal horizontal field (shorter‐wavelength, higher‐amplitude solution), we adjusted the damping level (isotropic variance factor) until we obtained a fit for the majority of the time series data to be within 2–3 *σ* (Figure S8). However, the histogram of the differences in vector magnitude revealed that there is a small population that has misfits much larger than 3  *σ* (Figure S9). Thus, we removed these large cGPS displacement outliers with more than a 3 *σ* misfit and re‐ran the inversions with the same isotropic variance factor. After doing this, we find that the SEUW equals 1.52 on average, which accurately reflects the fitting of the cGPS time series. An SEUW value of one typically represents an ideal isotropic variance (smoothing) factor (Beavan & Haines, 2001; Haines & Holt, 1993), but Beavan and Haines (2001) argued that an SEUW value of 1.5 is more realistic, owing to true error distributions in GPS observations. The damping level we choose matches the time series closely (Figure S8). By contrast the long‐wavelength solution typically underestimates the amplitudes of time series (Figure S8). The SEUW value of 6.37 for the long‐wavelength solution was also obtained after removing the same set of stations that were removed to obtain the final short‐wavelength solutions.

## 3 Results

### 3.1 Thirteen‐Year History of Transient Horizontal Deformation Inferred From cGPS: The Long‐Wavelength Solution

The modeled transient displacement anomalies, relative to North America reference frame (NA12), highlight remarkable seasonal periodic motions throughout much of the entire region containing the SAF, ECSZ, Great Valley and Sierra Nevada region. Animations of our 13‐year history of the long‐wavelength seasonal anomaly patterns of displacement, principal axes of strain, and the associated Coulomb stress changes throughout the plate boundary zone are available in the supporting information (Movies S1, S2, and S3). The large‐scale seasonal horizontal displacements influence the long‐wavelength strains in parts of Northern, Central, and Southern California, producing ± 10−20 × 10^−9^ dilatational strains and Coulomb stress changes on estimates of regional transform faults of ±1 kPa (Movies S1–S3).

**Figure 3:**
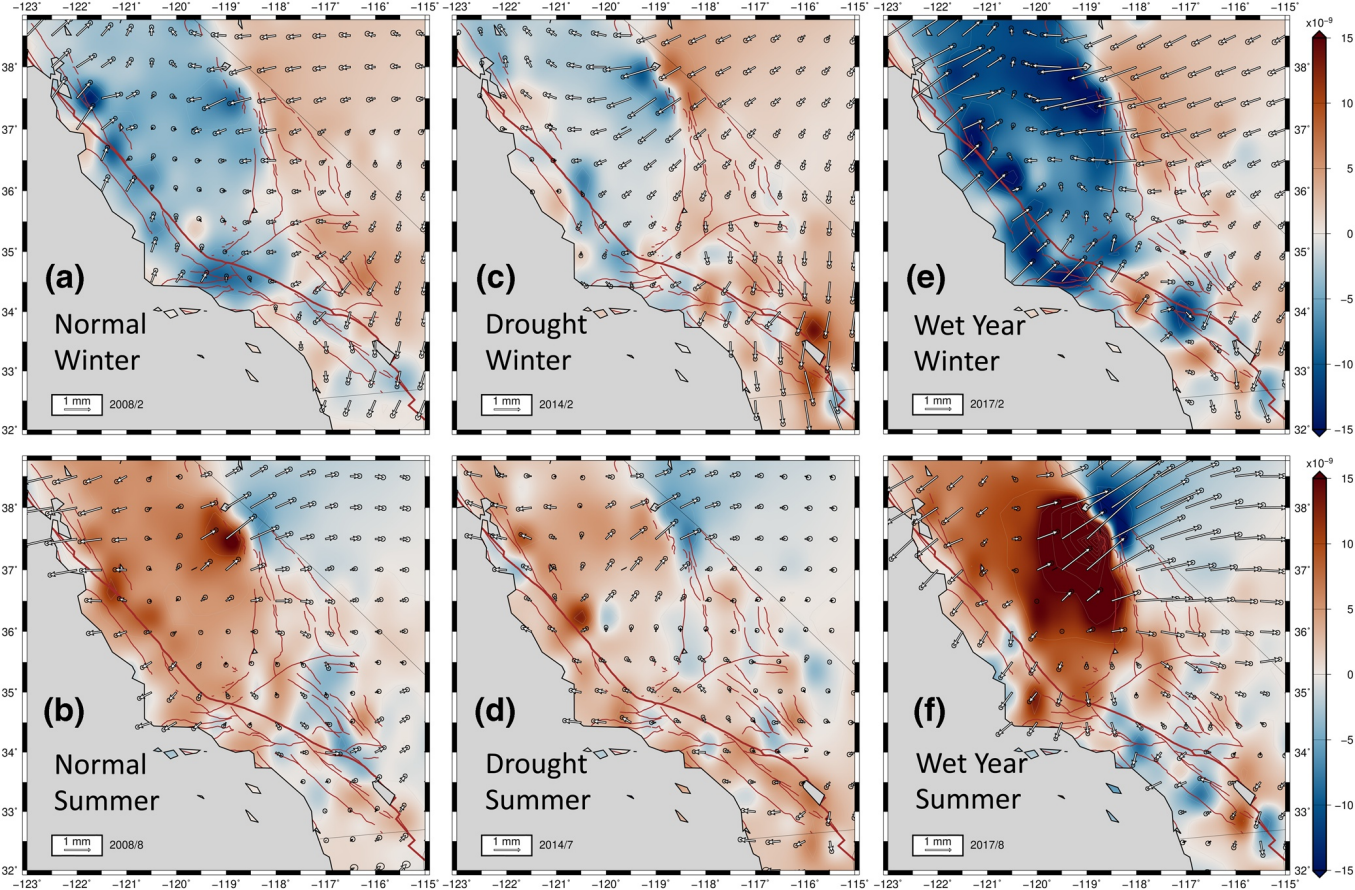
Model displacements relative to North America frame (NA12) obtained from smoothed fit to seasonal components of cGPS data processed by the Nevada Geodetic Laboratory (NGL), with dilatational strains plotted in background for winter (a) and summer (b) of 2008. We consider this year as a normal year. The model displacements representing the drought year deformation patterns are shown in (c) for winter and (d) for the summer of 2014. Note the weak winter pattern in the Great Valley (c) in comparison with a relatively normal winter of 2008 (a). Anomaly patterns for the drought period corresponding with the summer of 2014 (d). The deformation patterns during the heavy precipitation year of 2017 are presented for winter (e) and the following summer (f). The positive dilatation is extensional (red) and negative dilatation is contractional (blue). We present the corresponding solutions obtained using another level‐2 position time series processed by the GAGE facility (Central Washington University, [CWU]) in Figure S1.

**Figure 4:**
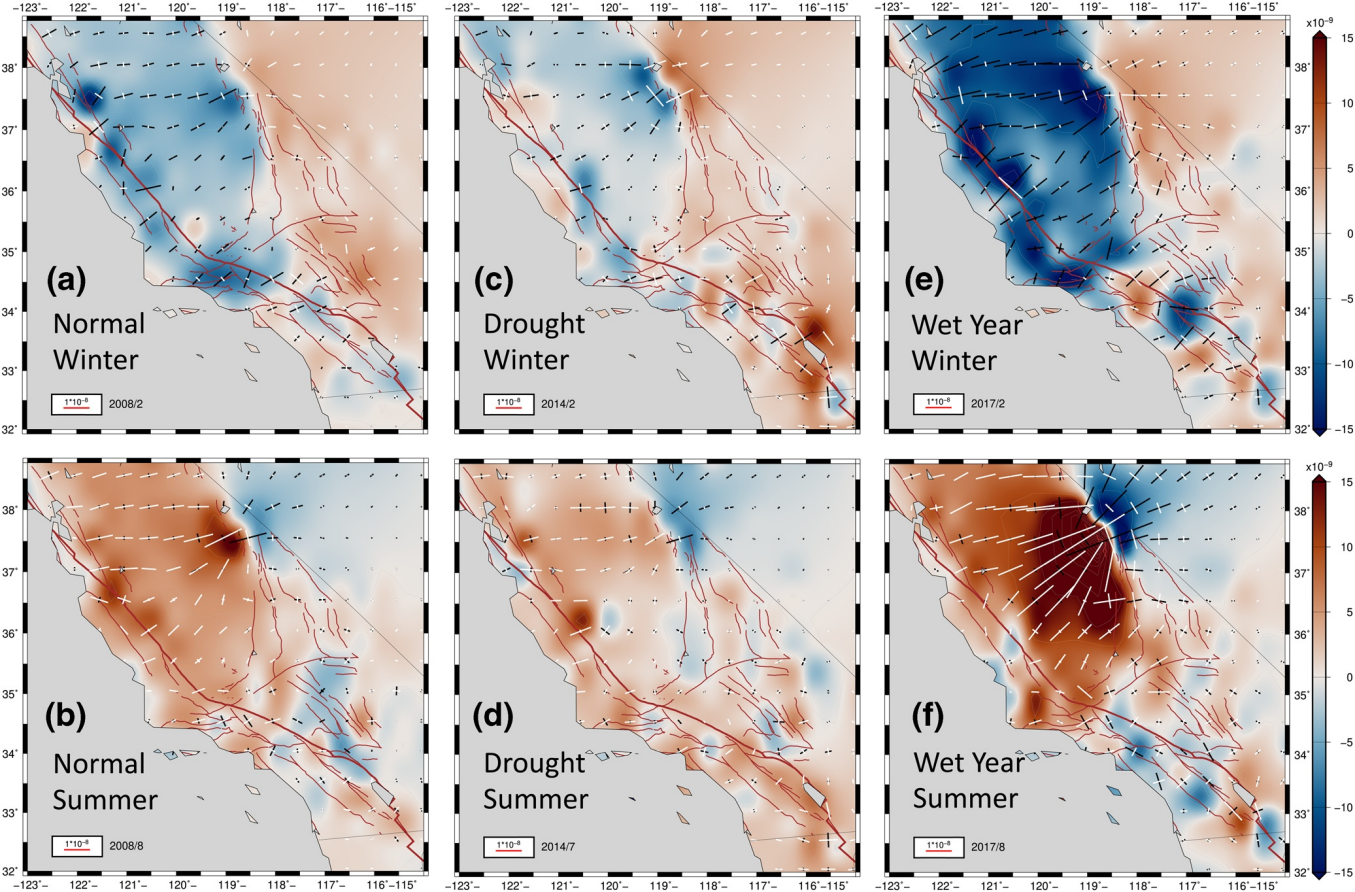
Model principal axes of strain from smoothed fit to seasonal components of cGPS data processed by NGL. The white axes are extensional; the black axes are contractional. The background is the same as in Figure 3. The positive dilatation is extensional. Each panel is equivalent to the same panel in Figure 3. Principal axes of strain for normal winter (a) and summer (b) of 2008. The drought year deformation patterns are shown in (c) for winter and (d) for the summer of 2014. The deformation patterns during the heavy precipitation year of 2017 are presented for winter (e) and summer (f). We present the corresponding solutions obtained using another level‐2 position time series processed by the GAGE Facility (CWU) in Figure S2. cGPS, continuously operating Global Positioning System; CWU, Central Washington University; NGL, Nevada Geodetic Laboratory.

During the winter, the Coast Range and the Sierra Nevada experience contractional dilatation (Figures 3a and 4a). Motions are up to 1–3 mm toward the Great Valley relative to North America frame. Much of the SAF and Sierra Nevada enter dilatational contraction in the winter. Southern California, west of the Mojave Desert, also undergoes contraction during the winter. During this same winter time interval, the ECSZ, east of the Sierra Nevada, and the Mojave, experience positive dilatation (Figures 3a and 4a). These regions generally undergo a Coulomb stress increase (unclamping on right‐lateral faults) during the winter, whereas the SAF experiences a decreased Coulomb stress change, resulting from the increased compressional normal traction along the SAF (Figures 3a and 4a).

During the summer (Figures 3b and 4b), a prominent zone of positive dilatation develops in regions around the SAF between 34°N and 37.5°N. The Great Valley and the Sierra Nevada also experience positive dilatational strains. The Coast Range and the Sierra Nevada displace a total of ∼1–3 mm outward from the Great Valley during the summer seasons. This transient divergent motion around the Great Valley and its spatial derivatives set up tensional normal tractions in summer along the SAF between 35.5°N and 37.5° N, resulting in positive Coulomb stress changes of up to 1 kPa there (Figures 3b and 4b). On the other hand, east of the Sierra Nevada, the Owens Valley north of latitude 36.5°N, and the Lone Pine fault zones generally experience a decrease in Coulomb stress change on right‐lateral strike‐slip faults, owing to a clamping of these shear zones associated with the contractional strains there (negative dilatation) in the summer (Figures 3b and 4b).

Another interesting feature is the dramatic variations in amplitudes of seasonal horizontal strain anomalies during drought periods (2012–2015) (Figures 3c, 3d, 4c, and 4d), as well as during the heavy precipitation in years of 2011, 2017, and 2019 (Figures 3e, 3f, 4e, and 4f). We define a precipitation year as the interval between November of the previous year to October (e.g., signals in the heavy precipitation year of 2017 are those between November 2016 and October 2017). In a precipitation year, winter strain signals are usually maximum between February and March, and the summer strain signals are usually maximum between August and September.

The winter dilatational strain patterns during the drought years (2012–2015) are diminished compared with those of normal winters (Figures 3c and 4c). The Great Valley, SAF, and Sierra Nevada generally experience less contractional strain during drought winters. The most diminished regions compared to the normal precipitation years are the Sierra Nevada and the Great Valley. During the drought summers (Figures 3d and 4d), similar magnitudes of extensional dilatation to that of normal years occur in regions around the SAF (Figures 3b and 3d), whereas the extensional dilatation in the Sierra Nevada and the Great Valley is less pronounced than in normal years (Figure 3d).

During the heavy precipitation in years of 2011, 2017, and 2019, the magnitude of winter dilatational strain patterns is significantly higher compared to those of normal winters, especially along the Sierra Nevada, the Great Valley, and along and adjacent to the SAF north of 34.5°N (Figures 3e and 4e). Contractional strains in these regions are typically higher by about a factor of two (Figures 3e and 4e) compared to normal years (Figures 3a and 4a). During the summers of heavy precipitation years, extensional dilatational strains are a factor of two to three larger in the Great Valley and the Sierra Nevada compared to normal years (Figures 3a, 3f, 4a, and 4f). Owing to the stronger extension along the Sierra Nevada and the Great Valley during the summers of the heavy precipitation years, the regions that lie east of the Sierra Nevada (north of 37°N) experience greater contractional dilatation than they do for normal or drought precipitation years (Figures 4b, 4d, and 4f).

### 3.2 Transient Horizontal Deformation Inferred From the UNAVCO Hydrologic Loading Model

Assuming that all the poroelastic and anthropogenic responses in cGPS are properly removed by following the procedure in Section 2.1, we compare the long‐wavelength horizontal strains inferred from cGPS with the horizontal strains associated with the UNAVCO hydrologic loading model (Puskas et al., 2017). We analyze the hydrologic loading time series using the same algorithm that we use to analyze cGPS horizontal data. The only difference from the treatment of real data is that we exactly fit the displacements from the hydrologic loading model, and there is no steady‐state reference model to subtract out, since this is a loading model only, and it does not contain a tectonic signal.

**Figure 5:**
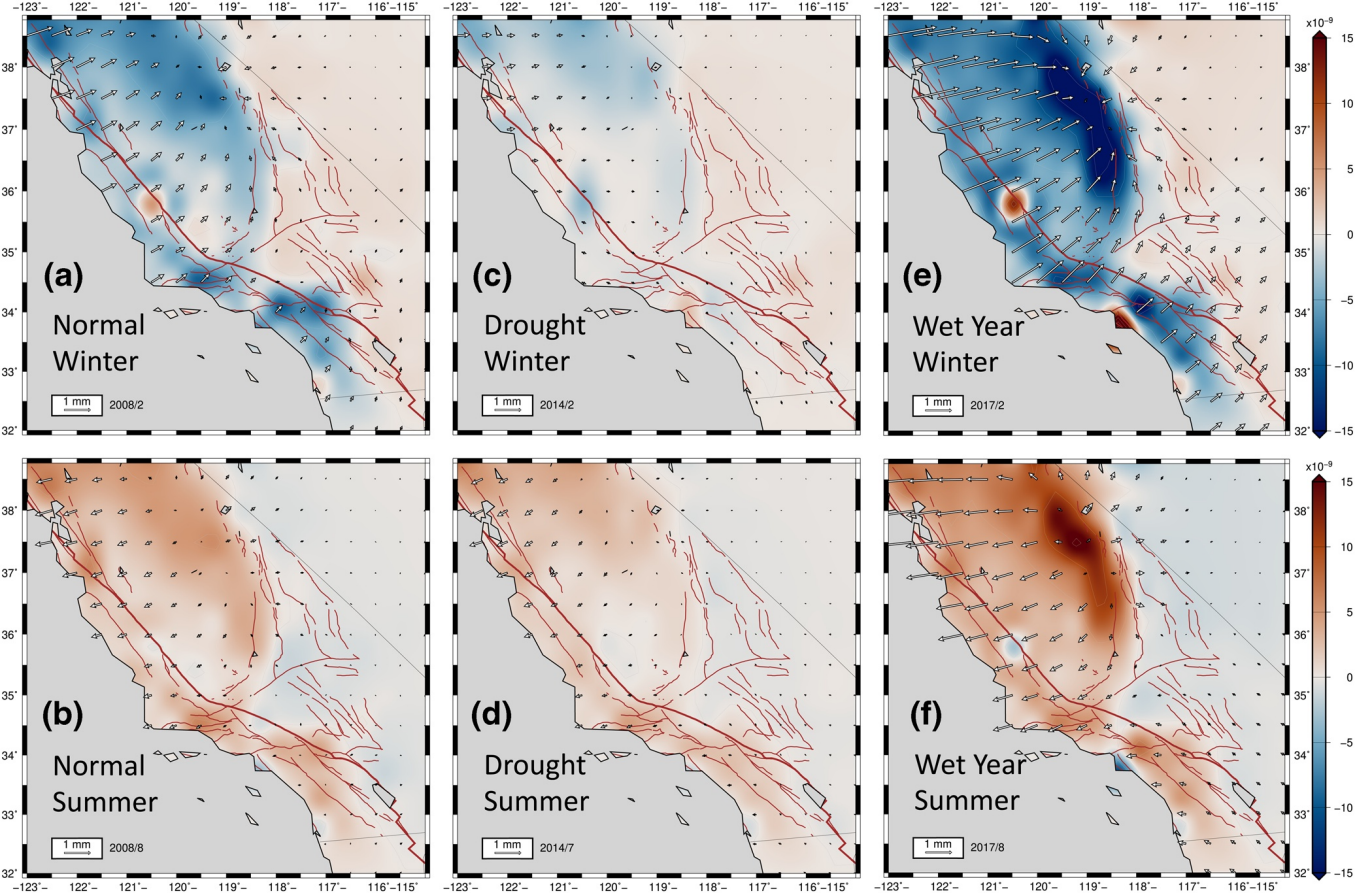
Model displacements relative to a fixed point at (38.1°N and 116°W) obtained from UNAVCO hydrologic loading model, with dilatational strains plotted in background for the normal winter (a) and summer (b) of 2008. Fixing the point approximates a “loading frame” because strains at this location in the UNAVCO hydrologic model are typically zero for all time frames. The hydrologic model displacements and deformation patterns for the drought year of 2014 are shown in (c) for the winter and (d) for the summer. Note the weak winter (c) and summer patterns (d) in comparison with the relatively normal winter (a) and summer (b) of 2008. The deformation patterns and motions during the heavy precipitation year of 2017 are presented for winter (e) and summer (f). The positive dilatation (red) is extensional. Note that the magnitude of the scale bar is the same as in Figure 3; therefore, direct comparisons of the seasonal patterns and the multi‐annual variations can be made.

The UNAVCO hydrologic loading model shows a seasonal negative dilatational strain along and adjacent to most of the SAF and the Sierra Nevada during the winter (Figures 5a, 5c, and 5e). During the summer, the dilatational strain patterns typically reverse, and the regions surrounding and along the SAF and the Sierra Nevada enter dilatational extension (Figures 5b, 5d, and 5f). The UNAVCO model has diminished dilatational strain during drought years (Figures 5c and 5d) and higher strains, relative to normal years, during heavy precipitation years (Figures 5e and 5f).

Dilatational strains from the UNAVCO model are only 10%–50% of the magnitude of the horizontal strains inferred from the cGPS. Overall, in spite of the smaller magnitudes, the long‐wavelength patterns for both solutions are generally similar for both summer and winter, and they show similar relative variations between normal, drought, and heavy precipitation years (Figures 3 and 5).

### 3.3 Transient Horizontal Deformation Inferred From Surface Water Estimates

To further investigate the physics behind the long‐wavelength transient deformation anomalies obtained using horizontal cGPS data, we take surface water estimates inferred from vertical cGPS and a composite hydrology model (Argus et al., 2017) and compute the elastic response as a horizontal displacement field. We use the modeling software ISSM‐SESAW v1.0 (Adhikari et al., 2016; Larour et al., 2012) to compute the horizontal component of the elastic response to vertical loading (Argus et al., 2017). The detailed procedure is in the supporting information (see Section 5 in supporting information S1). To compute the horizontal strain response to this elastic model, we apply the same procedure used to recover the horizontal strain from the UNAVCO hydrologic loading model.

**Figure 6:**
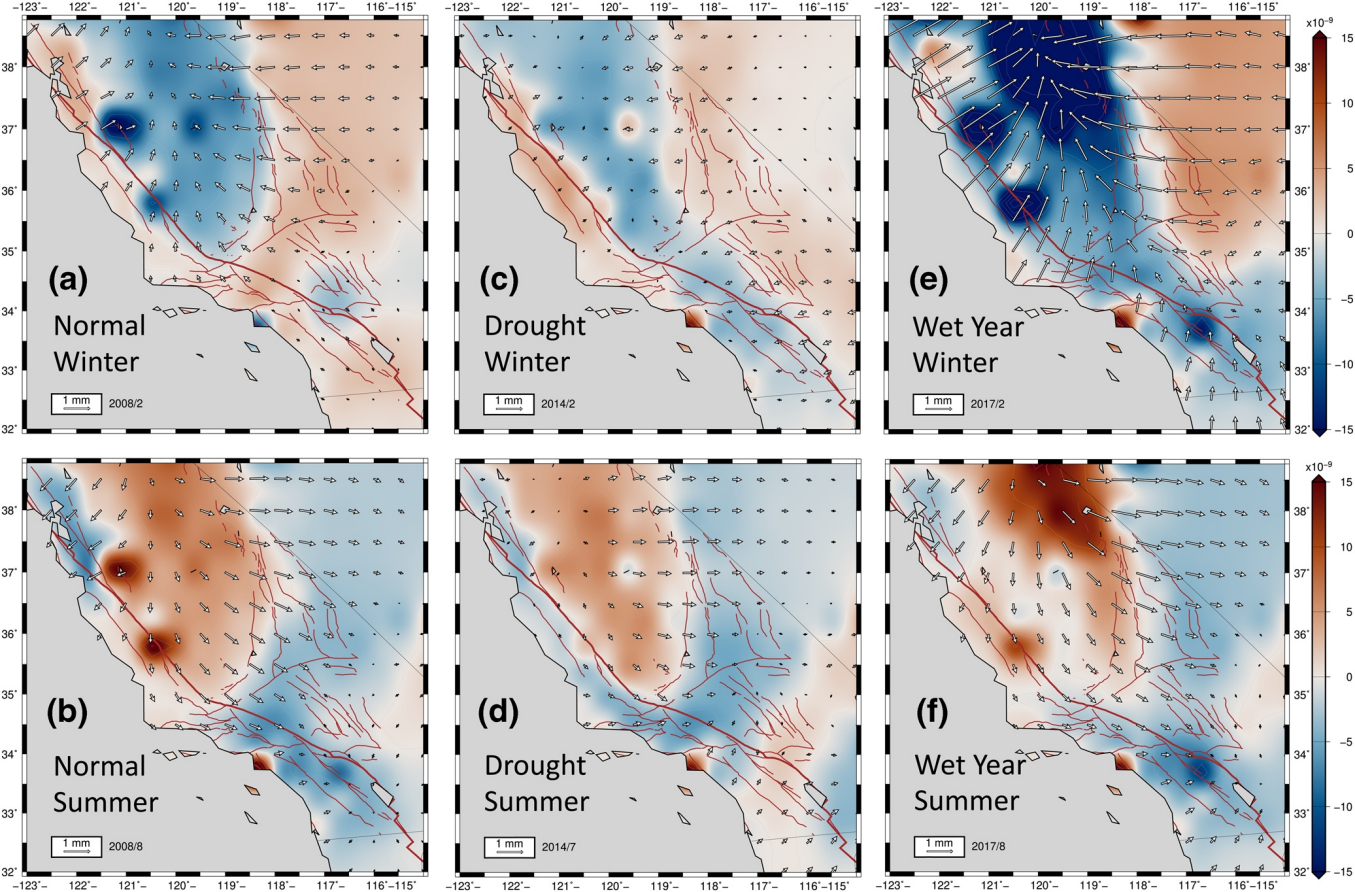
Model displacements relative to “loading” frame obtained from surface water equivalent estimates (Argus et al., 2017), with dilatational strains plotted in background for the normal winter (a) and summer (b) of 2008. The drought patterns are presented in (c) for the winter and (d) for the summer of 2014. Note the weak winter (c) and summer patterns (d) in comparison with the relatively larger signals for the normal winter (a) and summer (b) of 2008. The deformation patterns and motions during the heavy precipitation year of 2017 are shown in (e) for winter and (f) for summer. The positive dilatation (red) is extensional. Note that the magnitude of the scale bar is the same as in Figures 3 and 5; therefore, direct comparisons of the seasonal patterns and the multi‐annual variations can be made between Figures 3, 5, and 6.

During winters, the water load predictions (Argus et al., 2017) show negative horizontal dilatational strains along and adjacent to the SAF between 35°N and 37.5°N, as well as within the Great Valley and the Sierra Nevada (Figures 6a, 6c, and 6e). Opposite patterns of strain occur during most summers (Figures 6b, 6d, and 6f). The water load horizontal predictions also show the influence of drought (Figures 6c and 6d) and heavy precipitation years (Figures 6e and 6f). The comparison with the horizontal long‐wavelength solution demonstrates a similar agreement in pattern and magnitude for normal, drought, and heavy precipitation years.

### 3.4 Shorter‐Wavelength, Higher‐Amplitude Seasonal Variations in the Nontectonic Strain Field

**Figure 7:**
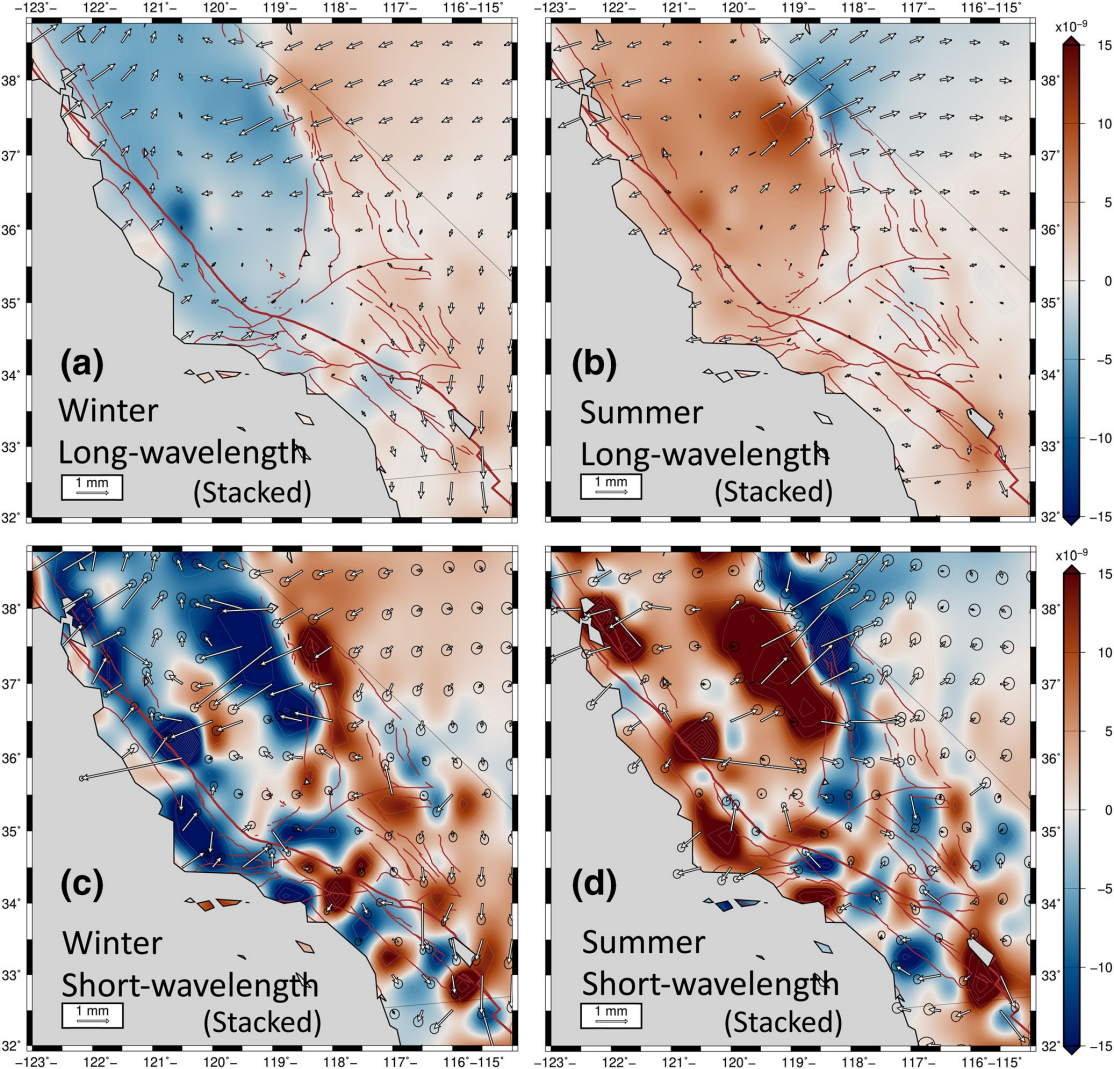
Comparisons between the long‐wavelength winter (a) and summer (b) solutions, and the shorter‐wavelength, higher‐amplitude winter (c) and summer (d) solutions. The stacked model displacements and dilatational strain representing winter averaged over January, February, and March between 2008 and 2019 except 2010, and summer averaged over July, August and September between 2007 and 2018, except 2010. The positive dilatation is extensional. 1 *σ* error ellipsoids are plotted.

It is important to note that the heavy damping (SEUW value of 6.37) that provided the long‐wavelength solution (Figures 3, 7a, and 7b) possibly smooths out and masks physically meaningful local features in the horizontal transient strain field. By applying a lower damping (which leads to the SEUW value of 1.52), we produce the higher‐amplitude, shorter‐wavelength horizontal transient strain solutions (Figures 7c and 7d). This lower damping leads to a closer fit to the cGPS (Figures 7 and S8), but larger posterior errors in the model strain field as mentioned in Section 2.4. However, the shorter‐wavelength, higher‐amplitude seasonal strain changes repeat reliably over space and time for the duration of our analysis. Because the anomaly patterns repeat every season, we stack the winter anomalies, as well as the summer anomalies, to obtain average seasonal signals (Figure 7). The errors for the stacked solutions are reduced by an amount roughly equal to one over the square root of the number of years (11 years). We exclude the solutions for 2010 owing to the M7.2 El Mayor‐Cucapah earthquake and its large co‐seismic and post‐seismic signals. Note that the 1 *σ* standard errors in displacement for the stacked long‐wavelength solution are small in comparison with the stacked shorter‐wavelength, higher‐amplitude solution (Figure 7). Nevertheless, the errors associated with the motions and transient strains for the shorter‐wavelength stacked solution are small enough such that the solution can be considered statistically significantly different from the long‐wavelength solutions.

Although the amplitude is higher, and the wavelength is shorter compared to the average long‐wavelength strains (40–100 km vs. ∼250 km), the average shorter‐wavelength, higher‐amplitude strain solution (Figures 7c and 7d) still shows large‐scale strain domains that match with the long‐wavelength solutions (Figures 7a and 7b). During the winter, for instance, the shorter‐wavelength, higher‐amplitude solution also predicts contractional strain in the Sierra Nevada and regions on and surrounding the SAF north of 34.5° N, but the amplitudes are two to five times higher, and more locally focused than the contractional strain predicted by the long‐wavelength solution in these regions (Figures 7a and 7c). The Great Valley south of 37°N experiences extension during the winter (Figure 7c). By contrast, the contractional dilation appears to be relatively evenly distributed across the SAF, Great Valley, and Sierra Nevada in the long‐wavelength solution (Figure 7a). In the same season (winter), much of the Owens Valley, southern ECSZ, and Mojave Desert experience two to three times larger dilatational extension than for the long‐wavelength strain field (Figures 7a and 7c).

During the summer, the shorter‐wavelength, higher‐amplitude strain patterns are, in general, opposite to the winter strain patterns (Figures 7c and 7d). Extensional strains develop in the Sierra Nevada and regions on and adjacent to the SAF north of 34.5°N, whereas the entire Owens Valley experiences dilatational contraction during the summer (Figure 7d). The strain contrast between the Sierra Nevada and the regions east of the Sierra is more pronounced in the shorter‐wavelength, higher‐amplitude solution. Note that shorter‐wavelength, higher‐amplitude summer signals are not often exactly antisymmetric to the corresponding winter signals in the same precipitation year, implying that the shorter‐wavelength solution cannot be solely explained by seasonally varying surface hydrologic loading (see Sections 4.2 and 4.3).

To statistically show that the shorter‐wavelength solution is different from the long‐wavelength solution, we plot two probability histograms of differences in the magnitudes of model and observed displacement vectors and the angular misfit between them (Figure S9). We infer that the long‐wavelength solution is missing real signal based on these histograms, along with the large differences in SEUW values (1.52 vs. 6.37). We find that the long‐wavelength solution misfits about 65% of the horizontal cGPS by more than 2 *σ*. This may imply that the optimal solution contains an additional transient strain field that is superimposed on a long‐wavelength elastic signal, which we infer to be directly associated with hydrologic loading. Evaluation of the ratio of the sum of squares of the differences between the long and short‐wavelength solutions and the sum of squares for the short‐wavelength solution alone, shows that these two solutions are 85% different (see Section 10 in supporting information S1).

The higher‐amplitude, shorter‐wavelength signal is not as well resolved as the long‐wavelength signal, reflected in the higher posterior errors. Nevertheless, the short‐wavelength seasonal anomalies repeat reliably in space and time. The checkerboard resolution test suggests that shorter‐wavelength anomalies (with length scales of ∼1° in longitude) can be recovered reasonably well (Figure S6) in regions adjacent to the SAF, within the Sierra Nevada south of 38°N, Mojave Desert, and the ECSZ. However, the Great Valley between 35.5°N and 36.5°N and the Sierra Nevada between 38°N and 39°N are not as well resolved. This is a function of cGPS station spacing and station distribution.

## 4 Discussion

### 4.1 Link Between Precipitation Patterns and the Horizontal Strain Anomaly Patterns in the Long‐Wavelength Solution Inferred From cGPS

The spatiotemporal similarities between the long‐wavelength solution and the hydrologic loading models (Argus et al., 2017; Puskas et al., 2017) support the hypothesis that the large‐scale (long‐wavelength) seasonal horizontal motion and strain of the crust within the plate boundary zone are a result of the solid Earth's elastic response to surface water load variations.

Both of the hydrologic loading models predict annual periodic strain patterns as shown in Section 3, which involve contractional‐dilatation during winters and extensional‐dilatation during summers in regions around the SAF, the Sierra Nevada, and the Great Valley that are similar to strain patterns from our horizontal long‐wavelength model inferred from cGPS. During the late winter, which is a wet season, the total water storage on the surface in California is maximum, leading to subsidence of the crust beneath peak loads (Argus et al., 2014). This negative vertical motion is recorded by vertical cGPS. Wahr et al. (2013) showed that the horizontal elastic response to a mass distribution on the surface (Farrell, 1972) is toward the center of the mass load. Our seasonal horizontal displacement field and its corresponding long‐wavelength contractional dilatation during the winter, therefore, can be explained by the elastic response to the surface water loads on and surrounding regions near the SAF (southern Coast Ranges), the Sierra Nevada, and the Great Valley. By contrast, during the summer, which is a dry season, the crust in California undergoes uplift because of loss of surface water load (Argus et al., 2014). The horizontal response to a loss of a mass distribution on the surface involves motions away from the center of the mass (Wahr et al., 2013). The extensional dilatation found in our long‐wavelength solutions during the summer periods can be interpreted as a consequence of the loss of surface hydrologic loads.

Comparison of the hydrologic loading models with our long‐wavelength horizontal transient solution reveals that year‐to‐year variations of transient strain patterns can be understood in the context of the history of precipitation over the last 13 years in California. For instance, we infer that the diminished winter signals during the severe drought are due to smaller surface water loads, and the enhanced winter signals of 2017 indicate the double‐stacked snow in the Sierra Nevada and abundant surface water in the Great Valley and in regions surrounding the SAF. The summer of 2017 presents the largest extensional dilatation pattern in the entire Great Valley, the Sierra Nevada, and in regions around the SAF between 39°N and 35°N. This large extension can be interpreted as an elastic rebound following removal of the heaviest surface loads in 2017. Other heavy precipitation years of 2011 and 2019 show similar patterns of strain to those in 2017.

After the El Mayor‐Cucapah earthquake, we detect a change in the patterns of strain in the long‐wavelength solution. The overall variations in the long‐wavelength strain anomalies in California following the post‐seismic relaxation are minor and limited in the regions south of latitude 34°N (Figure S10). This change is not observed in the hydrologic loading models (Argus et al., 2017; Puskas et al., 2017; Figures S11, S12).

### 4.2 Discrepancies Between the Long‐Wavelength Solution and the Shorter‐Wavelength, Higher‐Amplitude Solution

We now speculate on the origins of the shorter‐wavelength transient strain signal that appears to be superimposed on the hydrologic signal, which is characteristically long‐wavelength. The first possibility is that the methods that extract hydrologic loading signals from cGPS data are biased to only include a longer‐wavelength signal. Argus et al. (2017) inverted vertical cGPS data for the surface water estimates using the methodology of Argus et al. (2014). In their bounded‐value least squares algorithm (Stark & Parker, 1995), Argus et al. (2014) introduced a roughness factor, which determines how much a water equivalent thickness in a pixel can vary with the other values in neighboring pixels. Argus et al. (2014) settled on a roughness factor of 3. A smaller value of the roughness factor allows the model water thickness to vary more, but it would result in an unreliable water thickness model due to the uncertainties in the cGPS data (Argus et al., 2014). Nonetheless, this smoothing process involved in their inversion may filter out real shorter‐wavelength, higher‐amplitude components of surface water loads and the associated horizontal strain anomalies.

The second possibility is that there are inherent limitations in using only the vertical time series for inferring water load distributions. Wahr et al. (2013) argued that the use of horizontal displacements for large complex load distributions could provide unique information that the vertical data itself cannot provide. Although the magnitude of the vertical Green's function response for a load is two to three times larger than horizontal responses, the horizontal components can be more sensitive to determine the geometry of the loading distribution. This is because the horizontal elastic responses are expressed as a vector sum of each horizontal response from surrounding loads, whereas the vertical fields are defined as a simple scalar sum of the surrounding loads. Following this logic argued by Wahr et al. (2013), Adhikari et al. (2017) revealed glacial mass transport waves in Greenland using additional constraints provided by horizontal GPS signals, which the vertical GPS signals, alone, could not detect. Thus, by incorporating the horizontal observations as additional constraints for water load distribution, it may be possible to explain the shorter‐wavelength, higher‐amplitude strain signals as an elastic signal linked to hydrologic loading.

The third possibility for explaining at least a component of the shorter‐wavelength, higher‐amplitude seasonal strain anomaly pattern is that there are additional surface loading processes such as thermoelastic loading (e.g., Ben‐Zion & Allam, 2013). Moreover, poroelastic phenomena may significantly perturb the state of the crustal strain field (e.g., Amos et al., 2014; Argus et al., 2005; Carlson et al., 2020; Chaussard et al., 2014; González et al., 2012; King et al., 2007). Although we eliminate some stations affected by poroelastic processes (Section 2.1), the effects of poroelastic strains have possibly not been completely eliminated from the data set. Atmospheric pressure (e.g., van Dam and Wahr, 1987) is not a likely explanation for the large differences between the short‐wavelength, higher‐amplitude and long‐wavelength solutions because the signature from atmospheric pressure is generally small compared to estimates from long‐wavelength hydrologic loading models (Johnson et al., 2017b).

We present the residual strain field between the long‐wavelength and the shorter‐wavelength, higher‐amplitude solutions in the supporting information (Figure S13). The residual highlights the amplified signals in summer and winter along regions near the SAF north of 35°N, within the Sierra Nevada, as well as east of the Sierra Nevada within the ECSZ. The differences also highlight an extensional dilatational strain anomaly pattern in winter within the San Gabriel Valley (SGV) region that is not antisymmetric with contraction in the summer (e.g., primarily large in the winter only). We looked into the source of this anomaly and found dramatic extension primarily within the heavy precipitation winters of 2011, 2017, and 2019. King et al. (2007) showed that this expansion is a response to large amounts of aquifer recharge in the SGV during the heavy precipitation winters of 2004 and 2005. Their proposed mechanism appears to explain the large dilatational anomaly that appears in the difference plot in Figure S13. To further investigate this, we stacked 2011, 2017, and 2019 winter signals and compared them with the stack of drought winters of 2012, 2013, 2014, and 2015 (Figure S14). This comparison verifies that expansion of the SGV is magnified during heavy precipitation winters, and is being captured by our shorter‐wavelength, higher‐amplitude solution, which implies that some significant poroelastic signals remain in the data.

### 4.3 Influence of the Long Valley Caldera on the Strain Anomalies

The Long Valley Caldera (LVC) is a part of the region of our analysis (Figure 1). Therefore, volcanic activity could affect our non‐steady‐state strain solution (e.g., Hammond et al., 2019; Ji et al., 2013; Klein et al., 2019). Some expansion of the LVC is present in the steady‐state model that we subtract out (Figure S5). Therefore, it is only likely that we would detect LVC transients if they vary from our reference model. Hammond et al. (2019) found that an enhanced multiannual uplift and horizontal extensional dilatation occurred in the LVC during the drought years. They suggested that the dilatation observed during drought years was caused by magmatic inflation owing to lower pressure from the lower surface loads during drought. Our shorter‐wavelength, higher‐amplitude solution shows a positive dilatational signal in the LVC region during the drought summers of 2012, 2013, 2014, and 2015, in accordance with the findings of Hammond et al. (2019). This dilatational signal is unusual in that it was only observed during drought summers. In all other years involving normal and heavy precipitation, the summer signal in the LVC is a strong contractional dilatation. The reason for this is that during normal and heavy precipitation years, the unloading of the Sierra Nevada snowpack causes dilatational extension in the Sierra Nevada, and dilatational contraction in the summer in regions just east of the high topography of the Sierra Nevada. However, this rebound pattern is particularly weak during the drought summers, and thus the dilatational patterns in the LVC region remains positive, owing to the volcanic expansion that was also enhanced above normal during the drought (Hammond et al., 2019).

We also stacked the drought summer patterns in LVC (2012–2015) and compared with stacks of summer signals from the heavy precipitation years (Figure S15). These comparisons confirm that the shorter‐wavelength, higher‐amplitude signals are picking up the more rapid expansion of LVC during drought summers (Hammond et al., 2019). This difference plot (between the long‐wavelength solution and the shorter‐wavelength solution) also shows the influence of the dominant contractional dilatation signal that arises during summers following heavy precipitation winters (Figure S15).

The winter pattern is typically opposite, involving dilatational contraction during snowpack in the Sierra Nevada and dilatational extension east of the Sierra Nevada. Thus, the LVC region is the only area that experienced year‐round dilatational extension east of the Sierra Nevada during the drought of 2012–2015.

Because of the damping, the long‐wavelength solution does not show the local anomaly in the LVC regions, but rather the large‐scale, dominant hydrologic signal of normal, drought and heavy precipitation years (Figure 3). Note that the larger positive anomalies in the summers of heavy precipitation years (e.g., the summer of 2017; Figure 3f) are attributable to the greater short‐term (4‐month) rebound of the elastic crust, after being more flexed by the heavier surface water loads in the previous winter (e.g., the winter of 2017; Figure 3e).

The 4‐month moving time‐window treatment (Kraner et al., 2018) is not suitable for showing a multi‐annual trend pertaining to the drought signal observed by cGPS. Our method, however, does show the short‐term signal related to the drought, which is the smaller magnitude of the seasonal deformations due to less water on the surface during drought times. We also note that the 4‐month window in the shorter‐wavelength, higher‐amplitude solution is able to capture the LVC expansion during drought summers. To investigate the multi‐annual water loss during the severe drought (Argus et al., 2017; Borsa et al., 2014; Hammond et al., 2019), it is necessary to use the absolute magnitude of the position time series with respect to a reference time (e.g., Argus et al., 2017; Klein et al., 2019), instead of the moving time‐window approach.

### 4.4 The Sharp Transition in Horizontal Strain Anomalies Between the Sierra Nevada and the Basin and Range Province

Another interesting feature in the horizontal transient strain solution is the sharp transition, or sign change in dilatation, between the Sierra Nevada and the western Basin and Range Province for both winter and summer peak signals (Figures 3 and 7). A similar sharp transition in patterns of vertical component velocities between the Sierra Nevada and the Basin and Range Province has been reported over multiannual time periods using GPS (Hammond et al., 2016). Hammond et al. (2016) suggested that the sharp transition shown in their GPS imaging of the vertical velocity field can be attributed to a contrast in the strength of the lithosphere between the Sierra Nevada and the western Basin and Range Province, which may lead to a focusing of tectonic uplift in the southern Sierra Nevada. This may be true. However, the sharp transition in horizontal dilatation that we demonstrate in both the hydrologic loading models (Figures 5 and 6) and in the models inferred from cGPS (Figures 3 and 7) is likely dominated by the horizontal elastic response to variations in vertical loading.

There are two primary factors influencing the sharp transition in horizontal dilatational patterns. The first factor is the steep gradient in hydrologic loading in going from the Sierra Nevada (high equivalent water loads) into the western Basin and Range Province (lower equivalent water loads). The second factor is related to the horizontal Green's function response to a disk load (Adhikari et al., 2017; Wahr et al., 2013). This response involves a sign change for the derivative of the horizontal displacement at the edge of the disk load (Figure S16). By contrast, the vertical Green's function response involves continuous derivatives (with no sign change) with peak displacement at the disk load center that gradually diminishes with distance away from the center of the load (Figure S16). Thus, the sign change in dilatation that occurs at the boundary between the Sierra Nevada and the Basin and Range Province is the expected horizontal response to the high water loading in the Sierra Nevada in winter and the unloading in summer. That the response to the hydrologic loading models predicts the sharp boundary at the edge of the Sierra Nevada, observed in horizontal cGPS field, adds to our confidence in this interpretation.

Although the transition in horizontal strain in the long‐wavelength model can be explained by a loading response on a uniform homogeneous elastic Earth (Figures 3, 5, and 6), the shorter‐wavelength solution shows much higher amplitudes of strains on either side of the sharp boundary (Figure 7), which are underpredicted by the hydrologic loading models. It is possible that the lateral rheological transition at the edge of the Sierra Nevada, discussed in Hammond et al. (2016), may be responsible for enhancing the amplitudes of the strains, but this remains to be tested using numerical modeling approaches that allow for such lateral changes in rheology.

### 4.5 Implications for Time‐Variable Seasonal Fault Hazard

Our method can be used to monitor loading‐driven stress changes on vertical faults over space and time. Kreemer et al. (2018) showed that there is a correlation between long‐wavelength horizontal seasonal stress anomalies (inferred from cGPS) and the rate of seismicity in California, especially in northern California. Their results suggest more main shocks (m ≥ 2.5) occurred in northern California during the summer than during the winter.

We first use the long‐wavelength solution to quantify Coulomb stress changes on the right‐lateral vertical fault structures (Movie S3). Readers are referred to the supporting information (Section 1 in supprting information S1) for the calculation of Coulomb stress change on right‐lateral vertical faults (Figure S17). We showed earlier that this long‐wavelength solution is closely correlated with the Earth's expected elastic response to the distribution of the water loads in California. This large‐scale transient stress model predicts positive Coulomb stress change along the SAF during the summer, while negative Coulomb stress changes generally develop along the ECSZ. During the winter, the signal is opposite. Based on this model, we generally expect slightly higher seismicity rates along the SAF and reduced seismicity along the ECSZ during the summer. During the winter, we also generally expect the opposite pattern of reduced seismicity rates along the SAF and higher rates along the ECSZ. We also show that these seasonal stress changes vary in magnitude over years. For example, during the heavy precipitation years of 2017 and 2019, the amplitudes of the predicted Coulomb stress changes increase significantly. In the future, it should be possible to consider these multiannual stress magnitude variations in studies of statistics of earthquake populations, in addition to what has been done before using the seasonally averaged variations (e.g., Johnson et al., 2017a; Kreemer et al., 2018). Based on our model, we expect a larger population of earthquakes along the ECSZ during the heavy precipitation winters owing to the larger magnitude of stress change there in comparison with drought winters. Likewise, the SAF during the heavy precipitation winters should have a lower seismicity rate in comparison with normal or drought winters, owing to the larger contractional dilatation strains that yield negative Coulomb stress changes.

**Figure 8:**
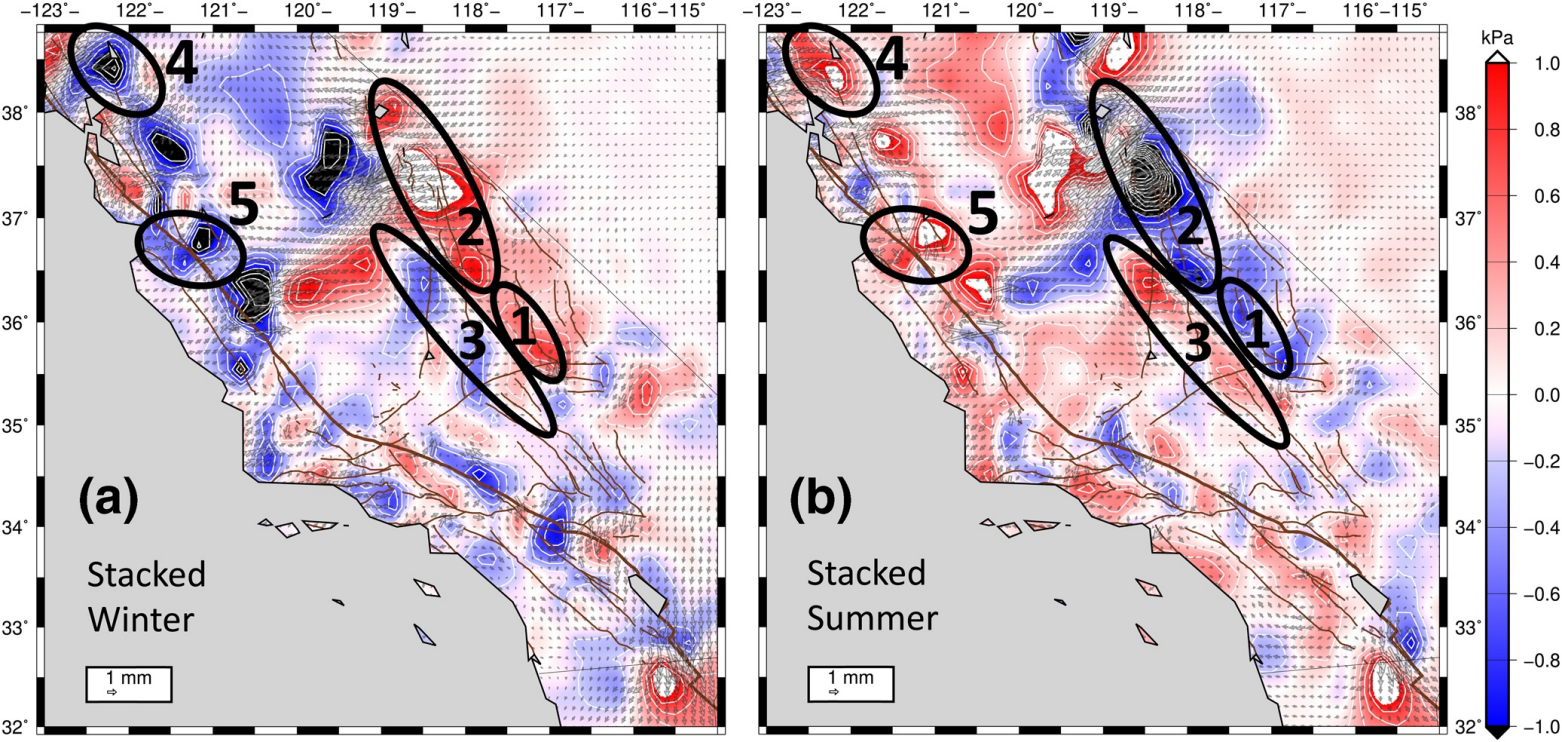
Stacked shorter‐wavelength, higher‐amplitude Coulomb stress changes on right‐lateral strike‐slip faults for winter (a) and summer (b). The labeled ellipses indicate examples of right‐lateral fault zones where we find notable seasonal strain anomalies and the associated stress changes. See text in Section 4.5 for description of fault zones in ellipses 1–5.

We next investigate the Coulomb stress changes associated with the reliable signals (areas with consistently repeating anomalies) found in our shorter‐wavelength, higher‐amplitude solution (Figures 7c and 7d). Kraner et al. (2018) reported that the 2014 M6.0 South Napa Earthquake of August was preceded by a Coulomb stress increase of 5.1 ± 1.6 *kPa* on right‐lateral faults in the region, caused by a short‐wavelength (∼50 km) seasonal positive dilatational strain anomaly that peaked that year, and every preceding year, in August. We identify several other regions that have reliable, repeating seasonally driven Coulomb stress changes (Figure 8). These regions, indicated by the ellipses in Figure 8, include: (1) The Panamint Valley, Hunter Valley, and Death Valley Fault zones—early winter positive Coulomb stress change anomalies and negative in summer; (2) northern Owens Valley, and Lone Pine fault zones—positive winter and negative summer Coulomb stress changes on right‐lateral faults; (3) Ridgecrest: Little Lake, and Airport Lake fault zones—positive Coulomb stress changes on right‐lateral faults in late spring/summer; (4) South Napa region, Green Valley Fault, Concord Fault zone—positive Coulomb stress change in late summer on right lateral faults; (5) Calaveras fault zone and San Andreas (Santa Cruz Mountains section)—positive Coulomb stress change in late summer on right‐lateral faults (Figure 8). Note that the 2019 Ridgecrest Earthquake Sequence, which was preceded by a positive Coulomb stress change in June, will have perturbed the patterns of a seasonal stress changes in the regions surrounding Ridgecrest. Future analysis using this technique will be able to quantify the evolution of stress changes, which will be a combination of seasonal loading and post‐seismic relaxation.

We have documented the time‐dependent seasonal stress changes (both long‐wavelength and shorter‐wavelength), and the possible impacts on seismicity rates need to be tested in future work. This would involve investigating correlations between the Coulomb stress changes in each region and seismicity rates in declustered catalogs (e.g., Zaliapin et al., 2008; Zaliapin and Ben‐Zion, 2013a, 2013b, 2016, 2020; Zhuang, 2006; Zhuang et al., 2002, 2004). Each of the regions identified above deserves its own detailed analysis because, while overall the patterns repeat reliably, there are subtle year‐to‐year variations in strain amplitude and strain orientation inside each of these regions. These variations from year‐to‐year are caused by fluctuations in surface water load patterns and magnitudes, along with other possible loading factors mentioned in Section 4.2.

Readers should keep in mind that our strain signals are accumulated strain anomalies over the previous 4 months. These are not strain rate anomalies, as we only analyze displacements. We also quantified 1‐month solutions. This is a short enough time interval such that these small strain increments are approaching the instantaneous strain change, or an approximation of strain rate after one divides the 1‐month strain by the fractional year that a month represents. In the supporting information, we show that the integration of the 1‐month solutions over 4 months generates identical solutions (Figure S18) to the 4‐month accumulated strain solution (Figure 3). When considering the influence of Coulomb stress changes on seismicity rates it may be more accurate to use the 1‐month solutions because they will contain a better temporal delineation of the evolution of stress change (Movie S5). It will be important to continue to monitor seasonal stress changes on faults going forward, with solutions updated each month. This will possibly enable a closer monitoring of time variable seismic hazards on fault sections that are known to experience repeating seasonal stress changes, which can vary in magnitude depending on the intensity and spatial distributions of hydrologic loading.

## 5 Conclusions

Based on the NOTA cGPS measurements of surface displacements, we quantify the 13‐year evolution of horizontal transient strain patterns between 2007 and 2019 within the plate boundary zone in California. Our long‐wavelength transient horizontal deformation model shows significant changes in strain over the 13 years. During the summer, a positive dilatation of 10‐20 × 10^−9^ develops in regions around the SAF and the Sierra Nevada, while part of the northern ECSZ and the western Basin and Range experience contractional dilatation. During the winter, the dilatational strain patterns are reversed. The Great Valley and the Sierra Nevada experience negative dilatation of 10‐20 × 10^−9^, and most of the ECSZ and the western Basin and Range Province undergo extension during the winter.

Seasonal strain patterns vary in magnitude and pattern over time. During the severe drought period between 2012 and 2015 (e.g., Argus et al., 2017), the amplitudes of the transient strains and displacements were diminished compared to those observed during the normal years. The average dilatations during the severe drought are only ∼46% (winter of 2014) and ∼45% (summer in the same year) compared to the normal‐year winter (2008). During heavy precipitation years of 2011, 2017, and 2019, we detect significantly enhanced variations in seasonal strain and displacement compared to those in normal years. The ratio of normal‐year (2008) to heavy‐precipitation‐year (2017) average dilatations along the Great Valley is ∼0.39 for the winter and ∼0.5 for the summer.

We investigate the link between the long‐wavelength strain anomalies and the solid Earth's elastic responses to the hydrologic loading on the surface. Using two hydrologic loading models (Argus et al., 2017; Puskas et al., 2017), we quantify the evolution of horizontal strain associated with the surface water loads between 2007 and 2017. Both hydrologic models predict, to first order, similar spatial and temporal seasonal patterns of strain during normal, drought, and heavy precipitation years, implying that the horizontal Green's function elastic responses to the surface water loads from the precipitation patterns in California can explain the first order long‐wavelength transient strain patterns observed by horizontal cGPS data. Our statistical test shows that the average correlation coefficients of dilatational strain between the long‐wavelength solution and the hydrologic models are 0.45–0.55. However, the high correlations require a heavy damping (with high SEUW values of 6–7) within the inversion of horizontal cGPS observations. That is, a large fraction of the horizontal cGPS (∼65%) are misfit by more than 2  *σ* error in the long‐wavelength solution.

By matching the cGPS data closely (with an optimal SEUW value of 1.52), we produce the higher‐amplitude, shorter‐wavelength horizontal transient strain solution with the larger posterior uncertainties in the model strain field. The shorter‐wavelength, higher‐amplitude seasonal strain changes repeat reliably over space and time for the duration of our analysis. Thus, we are able to obtain reliable average seasonal signals (Figures 7c and 7d) by stacking the winter and summer seasonal anomalies. While large‐scale strain domains match with the long‐wavelength solutions, the average shorter‐wavelength, higher‐amplitude strain solution shows additional amplification of seasonal anomalies in regions around the SAF, within the Sierra Nevada, and east of the Sierra Nevada within the ECSZ (Figures 7 and 8). The source of the local features in seasonal strain anomaly patterns are not as clear as the source for the long‐wavelength signals. Possibilities include coupled poroelastic‐elastic signal response to hydrologic loading, superimposed thermoelastic signals, or possibly lateral variations in elastic and viscoelastic properties of the lithosphere. The shorter wavelength, higher‐amplitude seasonal anomalies produce Coulomb stress changes on faults of the order 1–5 kPa. Although each overall anomaly is reliable from the standpoint of repeating each year, they show variations in detail that are dependent on whether it is a normal, drought, or heavy precipitation year. Focused studies on the time‐varying Coulomb stress change on faults, along with the potential impact on seismicity rates, are needed within several regions identified in this study.

## Supporting information

Supporting Information S1

Movie S1

Movie S2

Movie S3

Movie S4

Movie S5

## Data Availability

The data sets for this research are available in these in‐text data citation references: Blewitt et al. (2018), and Puskas et al. (2017); and the other data sets processed by Central Washington University are available at: https://www.unavco.org/data/gps-gnss/gps-gnss.html (accessed November 4, 2020).
