# Supplementary material for: Crustal Strain Patterns Associated With Normal, Drought, and Heavy Precipitation Years in California
Source: J Geophys Res Solid Earth. 2021 Jan 4;126(1):e2020JB019560. doi: 10.1029/2020JB019560 (PMC9285733; doi:10.1029/2020JB019560)
Supplement: Supplementary file 1 — Supporting Information S1 [file JGRB-126-0-s002.docx]

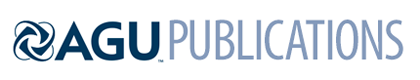


*Journal of Geophysical Research: Solid Earth*

Supporting Information for

**Crustal Strain Patterns Associated with Normal, Drought, and Heavy Precipitation Years in California**

Jeonghyeop Kim^1^, Alireza Bahadori^1^, William E. Holt^1^

^1^Department of Geosciences, Stony Brook University, Stony Brook, NY 11794

**Contents of this file**

Text S1

Figures S1 to S19

**Additional Supporting Information (Files uploaded separately)**

Movies S1 to S5

Text S1.

**1. Animations of thirteen-year history of seasonal variations in deformation (long-wavelength) and its associated Coulomb stress changes**

We select the panels in Figures 3 and 4 in the main text and provide three animations of displacement, strain, and the associated stress changes for the entire 13 year history. For the entire history of the periodic non-steady-state horizontal displacement, see Movie S1. Movies S2 and S3 show the entire history of the non-steady-state strain and associated Coulomb stress changes, respectively.

The procedure for computing Coulomb stress changes associated with seasonal strain variations is described in more detail in Kraner et al. (2018). Vertical strike-slip fault orientations within the plate boundary zone in California are inferred from our steady-state tectonic strain reference model defined by UCERF3 (Kraner et al., 2018), in which the no-length-change directions are defined from the strain field, following Holt and Haines (1993). We also resolve Coulomb Stress changes on fault geometries derived from another reference model obtained by averaging the 4-month accumulated strain solutions between the years 2007 - 2010. These two reference models (see Figure S5) are similar, as are the no-length-change orientations from them. Thus, the Coulomb stress changes resolved on fault orientations from these two models are negligible. For each of the reference models, we compute two possible no-length-change directions within each grid element and use these as the average expected orientations of fault structures that accommodate the field of steady-state strain (Holt and Haines, 1993; Holt et al., 2000). In most regions of interest, these no-length-change orientations are close to orthogonal and are consistent with vertical strike-slip faults along the SAF zone and Eastern California Shear Zone (ECSZ) (Figure S17) (Holt and Shcherbenko, 2013; Kraner et al., 2018). Following King et al. (1994), we resolve normal tractions and shear tractions onto these fault orientations to compute Coulomb stress changes on the inferred fault structures (Kraner et al., 2018, eq. 5, 6, and 7). We assume ﻿the effective coefficient of friction as 0.4 throughout the region (Toda et al., 2005). To convert our transient horizontal strains to stresses, we use a constant shear modulus of 30 GPa, assuming an elastic isotropic crust and zero Lamé parameter, λ, to approximate an incompressible elastic response.

**2. Models obtained using cGPS displacement data processed by GAGE**

The PBO (now NOTA) Level-2 product of position time series archived by UNAVCO used to be processed by two independent institutions (Central Washington University (CWU) and New Mexico Institute of Mining and Technology (NMT)) and merged together (Herring et al., 2016). This merged PBO data product is no longer available as of September 2018. Data processed by CWU, however, is still available for the entire thirteen years (from January 2007 to June 2019). The data products are available through UNAVCO (available at: ftp://data-out.unavco.org/pub/products/position) so that we solely use the CWU data set to produce our long-wavelength model. We find that the uncertainties in displacement product processed by GAGE are larger (about 5 times) than that processed by Nevada Geodetic Laboratory (NGL) (available at: http://geodesy.unr.edu/gps_timeseries/tenv/NA12/). We obtain similar long-wavelength seasonal transient strain model derived from the data processed by CWU, applying a smaller damping level for the same inverse methods described in the main text (*Section 2.6*). The smaller damping level is necessary, owing to the larger errors in the GAGE data (*Section 2.4*). The average SEUW of the long-wavelength solution obtained using the CWU data is 2.272 (The average SEUW of the solution derived from NGL is 6.37). The larger posterior uncertainties in the model displacements reflect the higher level of uncertainty in the GPS time series solutions provided by CWU.

Another difference between the two Level-2 position products is their reference frames. Time series positions processed by CWU and NGL are resolved in slightly different North America reference frames of NAM08 (Altamimi et al., 2012) and NA12 (Blewitt et al., 2013), respectively. We present our solution obtained using the data processed by NGL in NA12 North America reference frame. On the other hand, for the solution derived from CWU data, we solve for a best-fit rotation vector that aligns the vector field obtained from CWU with the field provided by the NGL in the NA12 North America reference frame. This enables a direct comparison of the two vector fields, but it does not impact the horizontal strain field obtained from the CWU solution. Movie S4 shows the rigid body rotation applied to the CWU solution for each time step to place it into a frame aligned with NA12. Here, we present displacement (Figure S1) and strain (Figure S2) solutions obtained using CWU data, which are equivalent to Figure 3 and Figure 4 in the main text. We also present the Coulomb stress changes inferred from CWU data (Figure S3), which can be compared to the results in Movie S3.

For both of our long wavelength solutions derived from the data products of CWU and NGL, we use the same number of NOTA stations for each time interval in order to avoid resolving different features in deformation caused by different distributions of the stations. For this purpose, we analyze vertical time series produced by CWU following the methodology in the main text *section 2.1* to distinguish and eliminate the stations suspected being affected by poroelastic or anthropogenic activities. We present the number of cGPS datasets that we employ to infer the long-wavelength horizontal transient model in California as a function of time (Figure S4). The number dramatically increased from the beginning of 2007 and reached over 500 by the January of 2008.

**3. Steady-state tectonic solutions**

When inverting cGPS data, we minimize the objective function (2) presented in Kraner et al. (2018), using a prior “observed” strain $e_{ij}^{obs}$ within each grid cell. This approach treats the observed strains as additional constraints on the inversion to the cGPS observation $u_{i}^{GPS}$. We obtain the “observed” strain $e_{ij}^{obs}$ using the UCERF 3 consensus GPS velocity field following Kraner et al. (2018). For the details, readers are referred to the supporting information written by Kraner et al. (2018) *(*See *section 1.0* in their Text S1*)*. Using equation (2) in the main text based on the UCERF 3 model, we find that transient displacements in the Parkfield and LA Basin regions often exceed 10 mm. This is unlikely to be a real non-tectonic signal accumulated over a short time period (4 month). As an alternative to the UCERF 3 model, we take a 3-year average of the long-wavelength transient horizontal continuous model field of displacement as our reference model $\bar{\Delta u} \left( x,y \right)$to perform the subtraction expressed in the equation (2). We find the new average reference model is close to the original reference model derived from UCERF 3 (Parsons et al., 2013; Field et al., 2014), while the signals near Parkfield and LA Basin slightly decrease in the new reference model (Figure S5). These reduced signals allow us to produce more realistic magnitude of non-steady-state transient signals (< 6 mm).

**4. Significance of post-seismic signals for the long-wavelength strain field inferred from cGPS**

We do not eliminate co-seismic and post-seismic signals. Our goal is to quantify the long-wavelength aspects of all non-steady-state components. We are interested in quantifying how post-seismic deformation adds or subtracts from the long-wavelength seasonal signal that we are capturing. The earthquake-driven signals can be large in comparison with seasonal hydrologic signals and they perturb or alter succeeding seasonal signals. For instance, the 2010 Mw 7.2 El Mayor-Cucapah (Baja California) earthquake of the April 4^th^ marks a pronounced change in behavior of long-wavelength seasonal patterns in southern California.

Before this large event, we find a systematic seasonal variation in displacement and strain in regions south of 34º N in southern California, although the periodic patterns are not as prominent as those found in regions to the north, including for the years following the El Mayor-Cucapah event. The southern part of ECSZ, within the Mojave, experienced extensional dilatation during the winter; the San Jacinto Fault zone and LA basin typically underwent dilatational contraction (Figure S10a). During the summer before April 2010, the overall patterns of the strain field in the same regions south of 34 ºN are opposite to winter (Figure S10b). During summer the southern part of ECSZ was typically in a state of contractional dilatation. The southern half of the San Jacinto Fault and most of LA basin, however, typically experienced extensional dilatation during summer.

After the El Mayor-Cucapah earthquake, we detect a change in the patterns of deformation in the same regions south of 34º N in the model obtained using cGPS data. Up to November 2015, the extensional dilatations are larger and more pronounced near the Salton Sea after the event regardless of seasons (Figure S10c, S10d). This extensional signal observed in our long-wavelength model is consistent with the post-seismic relaxation effects (e.g. Gualandi et al., 2019; Holt and Scherbenko, 2013), which has persisted at least through 2018. Gualandi et al. (2019) show that the post-seismic deformation has been observed more than 7 years and it is still ongoing. The changes in the long-wavelength deformation patterns before and after the El Mayor-Cucapah Earthquake, however, are limited in the regions south of latitude 34º N. The overall variations in large-scale patterns of strain anomalies in California following the to the post-seismic relaxation seem minor, especially in the region north of 34º N.

**5. Quantifying elastic responses to the surface water estimates (Argus et al., 2017) using the ISSM-SESAW software (Adhikari et al., 2016)**

The relationship between a displacement vector $\bar{u}$ at a station and surrounding surface loads can be expressed by

$\bar{u}= \int\bar{G}HdS$, (1)

where $\bar{G}$ is the Green’s function vector as a function of an arc distance and azimuth between the station and each surface load, parameterized by Legendre polynomials and load Love numbers, and H is a surface water equivalent (Farrell, 1972; Wahr et al., 2013; Adhikari et al.,2017). We perform the forward modeling calculation (eq. 1) to predict the horizontal elastic response to the surface load estimates at 1/8º intervals of latitude and longitude (Argus et al., 2017). We define kernel functions of horizontal elastic responses to disk loads of a constant water equivalent of 1 meter, using NASA Jet Propulsion Laboratory (JPL)’s modeling software ISSM-SESAW v1.0 (Adhikari et al., 2016) (Figure S16). The horizontal elastic response to a disk load on the surface (Farrell, 1972) is toward the center of the load, while the horizontal response to a loss of a disk load on the surface involves motions away from the center of the load (Wahr et al., 2013). When computing the kernel functions, we consider the area of each curvilinear grid where the water equivalent is estimated; each disk with 1-meter water equivalent has the same area as the corresponding 1/8ºx1/8º grid on the spherical surface. The radii of the disks slightly vary with a factor of cosine of latitude (Argus et al., 2014; Argus et al., 2017). For instance, the radius of a disk for a grid at latitude of 32.063º N is 7.142 km and the radius of a disk for a grid at latitude of 39.938º N is 6.793 km.

We use Preliminary Reference Earth Model (PREM) (Dziewonski and Anderson, 1981) in order to be consistent with Argus et al., (2017). Using the kernel functions, we generate the Green’s function vector for horizontal components and conduct the forward modeling calculation. For each location of stations, we set the farthest arc distance as 500 km, within which all the loads can contribute the horizontal displacements at the stations. We test the different limits of the farthest arc distance, and find that the model produced with the limit of 500 km shows the greatest similarities with our horizontal long-wavelength model. The latitudinal wavelength of the surface water estimates (Argus et al., 2017) are close to 100 km. Most of the variations in the surface loads occur in the Great Valley, the Sierra Nevada, the Klamath Mountains and the Coast Range, which have a loading width about 100 km. Wahr et al. (2013) argue that a distance exceeding 5 diameters from the edge of a single disc is typically too far to pick out the horizontal responses in GPS observations. Our choice of the farthest arc distance of 500 km corresponds to 5 disk diameters away from each of these major load regions.

The absence of surface water load estimates in the region south of latitude 32º N may cause some minor differences between the models. Using precipitation data obtained from 102 weather stations operated by Ministry of Agriculture and Water Resources of Mexico, Minnich et al. (2000) show average annual precipitation of about 30 cm along the mountain ranges north of 31º30′ N in Baja California, including the Sierras Juárez and the costal Sierra Juárez during the winter (November to May). A similar amount of annual precipitation is observed along the west flank of the San Pedro Mártir, which locates around latitude of 31º N in Baja California. About half of the peninsula, however, is arid, especially within the southeast regions off the mountain ranges. Only about 20% (5-6 cm) of water in winter is inferred by Minnich et al. (2000) to occur off the mountain ranges. Although the inclusion of water distributions along the mountain ranges in Baja California may modify the horizontal Green’s function responses for horizontal displacements in southern California, the amount of water is not likely large enough to significantly alter the displacement patterns within the hydrologic model for southern California.

**6. Different models of horizontal transient deformation derived using water estimates resolved on regular grids and a distribution of NOTA stations.**

We resolve the horizontal Green’s function responses to the surface load estimates (Argus et al., 2017) on regular grids of 0.1ºx 0.1º in the region of interest (Figure S19). Although the overall patterns of deformation are similar (contractional winter and extensional summer) we find that roughly circular dilatation anomalies along the San Andreas Fault, one near Parkfield region (~35.7º N) and another near latitude of 33.7º N (Figure 6), disappear in the solution computed using displacement output on a regular grid (Figure S19). In addition, we observe an enlarged dilatation signal in the Great Valley at the latitude of 38º N in the solution revolved on the regular grid (Figure S19), which we do not find in the other solution (Figure 6). These differences may indicate that there is minor spatial aliasing arising from the irregular distribution of the network of NOTA. However, the overall long-wavelength features of the field are preserved, regardless of whether a regular grid or the NOTA distribution of stations is used.

For the purpose of comparisons between the long-wavelength model inferred from cGPS and the two hydrologic loading models (Argus et al., 2017; Puskas et al., 2017), we present the loading solutions obtained using the horizontal Green’s function resolved in the location of NOTA stations (Figures 5, 6). Another notable difference between the models appears in Nevada, owing to the sparser NOTA network in the state compared to California. We find the spatial gradient of horizontal elastic responses in Nevada are more prominently resolved on the regular grid than in the case obtained using the locations of NOTA stations.

The a priori composite hydrologic model in the Great Valley (Argus et al., 2017) may also cause differences between the models. We compute the horizontal responses to the water equivalent estimates without the a priori model and compute the strain model, but the solution does not change much. Minor differences appear along the Great Valley near latitude of 36º N when we eliminate the a priori hydrologic model in the Great Valley.

**7. Definition of the Root Mean Square Error and the spatial correlation function between the model inferred from cGPS and the hydrologic loading models**

The spatial RMSE and correlation, *C,* for each 4-month epoch (a total of 150) are defined as

$RMSE= \sqrt{\frac{\sum_{i=1}^{n} \left( \theta_{i}^{GPS}-\theta_{i}^{loads} \right)}{n}}$ , and $C= \frac{\iint\theta^{GPS} \theta^{loads}dA}{\sqrt{\iint\theta^{GPS} \theta^{GPS}dA}\sqrt{\iint\theta^{loads} \theta^{loads}dA}}$ (2), (3)

where $\theta^{GPS}$ is the dilatational strain obtained using cGPS data and $\theta^{loads}$ is the dilatational strain inferred from the two hydrologic loading models. We compute average RMSEs and correlations over the thirteen years only using winter (February and March) and summer (August and September) months when amplitudes of seasonal strain anomalies are greatest. The correlation values and RMSEs from 2010 and 2011 are excluded because of the presence of significant long-wavelength post-seismic signal from 2010 Mw 7.2 El Mayor-Cucapah event in the model inferred from cGPS. Testing various damping levels (SEUW; see the main text *section 2.6*), we plot the average RMSEs and correlations as a function of SEUW (Figure 2).

**8. Time series of monthly average 4-month displacements**

We present observed time series of monthly average 4-month displacements for 10 stations across the region of interest (Figure S8). We make the comparisons presenting the long-wavelength solutions and the shorter-wavelength solutions with the observed horizontal time series. We choose the 10 stations out of the 181 stations that had been available from January 2007 to June 2019. The stations are P197; P224; P309; P301; P305; P571; P467; FGST; BKAP; and P612. Documented steps in the time series such as GPS antenna changes are removed (<http://geodesy.unr.edu/NGLStationPages/stations/>) .

**9. Checkerboard test**

As described in the main text, we perform a checkerboard test to show (1) the resolution of the network and (2) the effect of different damping levels. We generate two synthetic checkerboards: 2º x 4º and 2º x 1º grids, assigning alternating dilatations of $\pm30 \times{10}^{-9}$ and $\pm45 \times{10}^{-9}$to the long-wavelength checkerboard and the shorter-wavelength one, respectively. We then sample the corresponding horizontal displacements at the locations of the 678 NOTA stations that we use to obtain the long-wavelength strain solution presented in the main text. We first try to recover the two checkerboard patterns separately to show the resolution of the station distribution (Figure S6). We are able to recover much of the short and long-wavelength features across the region of interest using the horizontal displacement fields with Gaussian-distributed noise in a similar level to the errors in the observed monthly average cGPS displacements from the time series from NGL (~0.1mm). Second, we add the two synthetic checkerboards to simulate a “total” field that contains both the larger checkerboard distributions that are superimposed by the smaller checkerboards (Figure S7a). We sample a new set of horizontal displacements corresponding to the “total” field at the same locations of cGPS data. Using the synthetic displacements, with Gaussian noise added, we experiment with inversions using different damping levels to try to recover the “long-wavelength” input (distribution of larger checkerboards) as well as the “total” field (complete shape). We vary the damping level and calculate the correlations between the synthetic “total” dilatation and the recovered one, as well as the correlations between the synthetic “long-wavelength” dilatational field and the recovered one (Figure S7d). This test reveals that a heavy damping is required to extract the long-wavelength components from the “total” field (Figure S7b), while we need a smaller damping level to recover the “total” field (Figure S7c). This checkerboard test is analogous to our effort with the real data, and demonstrates that the gross shape of the larger checkerboard field can be recovered with the higher damping levels, while the test also demonstrates the ability of the network to recover the “true” or total field that contains the superposition of both the larger checkerboard pattern and the smaller checkerboard pattern.

**10. Quantification of the similarity between short and long-wavelength solutions**

To quantify the similarity between the short and long-wavelength solutions, we evaluate the sum of squares for the 7185 areas for the short-wavelength solution for all time steps (Beavan and Haines, 2001):

${SS}_{A}= \sum_{cells} [\frac{e_{11}^{2}}{Var\left( e_{11} \right)}+\frac{2e_{12}^{2}}{Var\left( e_{12} \right)}+\frac{e_{22}^{2}}{Var\left( e_{22} \right)}]$ (4),

where $Var\left( e_{ij} \right)$ represents the posterior model strain variance for that component of strain, $e_{ij}$. We also compute the difference between the optimal solution and the long-wavelength solution using:

$SS_{diff}= \sum_{cells} [\frac{\left( e_{11}-e_{11}^{'} \right)^{2}}{Var\left( e_{11} \right)}+\frac{2\left( e_{12}-e_{12}^{'} \right)^{2}}{Var\left( e_{12} \right)}+\frac{\left( e_{22}-e_{22}^{'} \right)^{2}}{Var\left( e_{22} \right)}]$ (5),

where the long-wavelength solution is represented by the prime. For all time steps the value for ${SS}_{A}$ varies between $5.6\times{10}^{3}-3.4\times{10}^{4},$ and the $SS_{diff}$varies between $4.7\times{10}^{3}-2.8 \times{10}^{4}$. The ratio between $SS_{diff}$ and ${SS}_{A}$varies between 0.82 – 0.86, with an average of ~0.85. This demonstrates that there is an 85% difference between the two fields. We also find a ratio of 0.83 for the stacked winter optimal to the stacked winter for long-period solution. This ratio is 0.82 for stacked summer solutions.

**
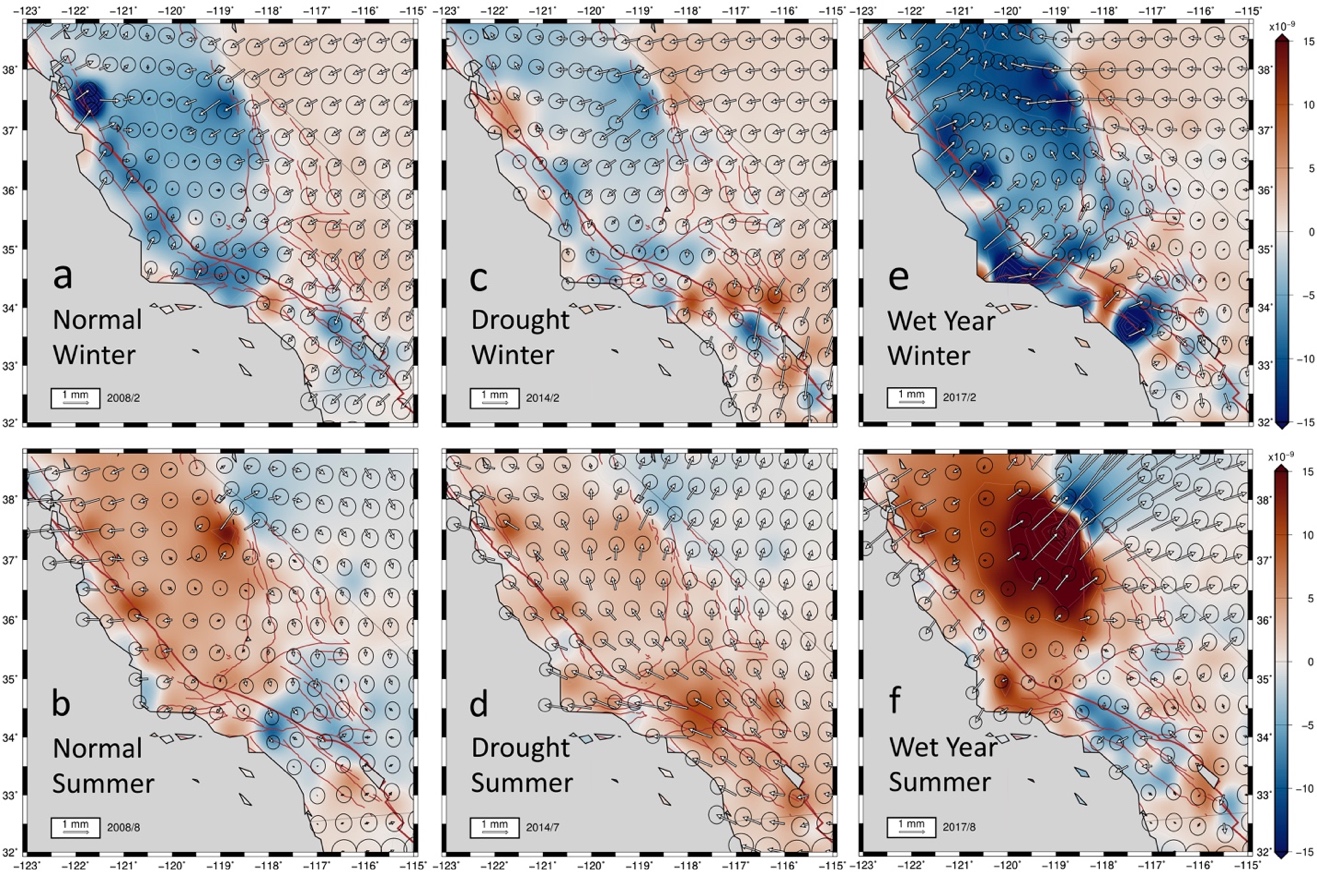
**

**Figure S1.** Model displacements relative to North America frame (NA12) obtained from smoothed fit to seasonal components of cGPS data processed by CWU, with dilatational strains plotted in background for winter **(a)** and summer **(b)** of 2008. Error ellipses represent one standard deviation. We regard this year as a normal year. The model displacements representing the drought year deformation patterns are shown in **(c)** for winter and **(d)** for the summer of 2014. Note the weak winter pattern **(c)** in comparison with a relatively normal winter of 2008 **(a)**. Anomaly patterns for the drought period corresponding with the summer of 2014 **(d)**. The deformation patterns during the heavy precipitation year of 2017 are presented for winter **(e)** and summer **(f)**. The positive dilatation (red) is extensional.


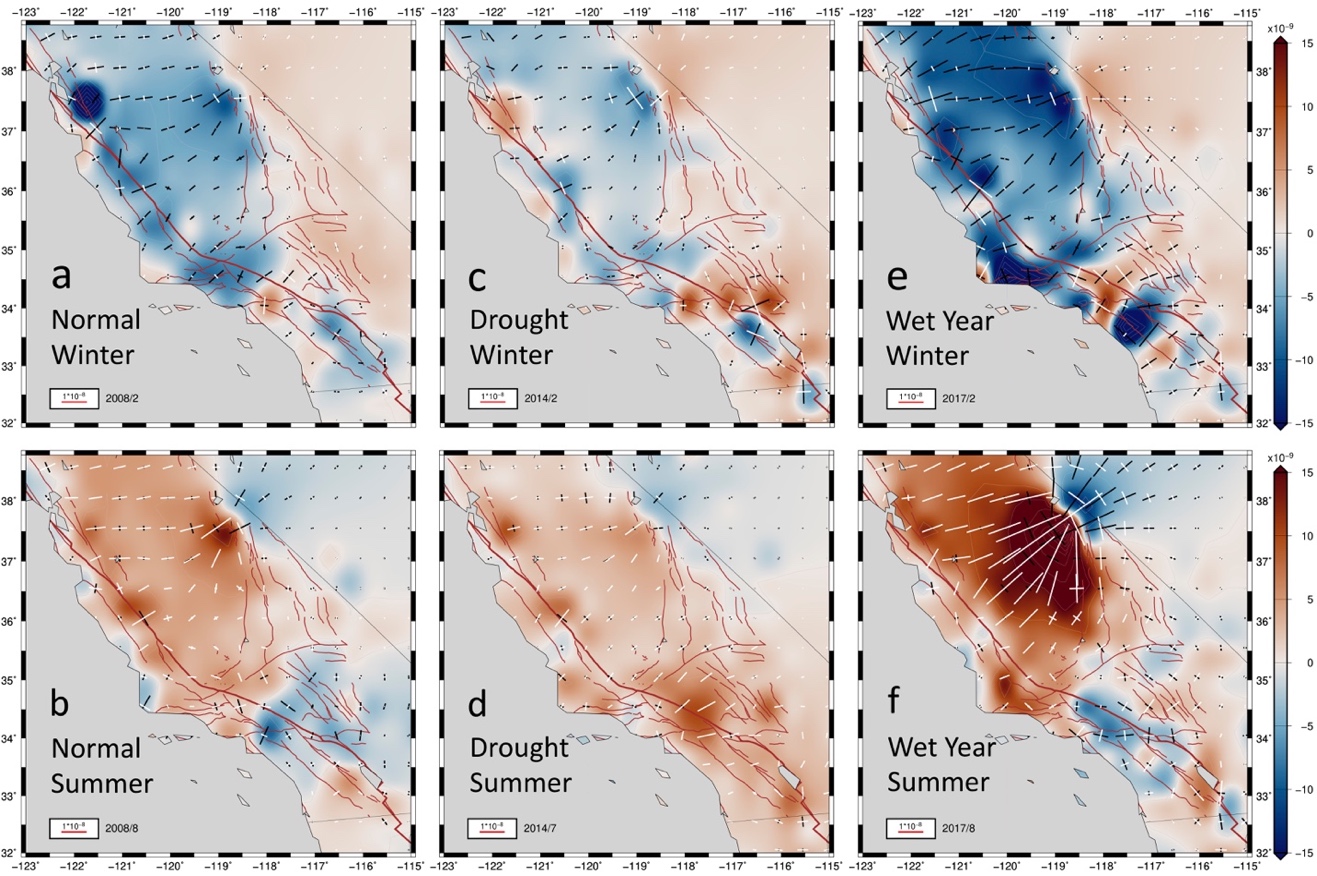


**Figure S2.** Model principal axes of strain from smoothed fit to seasonal components of cGPS data processed by CWU. The white axes are extensional; the black ones are compressional. The background is the same as in Figure 2. The positive dilatation is extensional. Each panel is equivalent to the same panel in Figure 2. Principal axes of strain for normal winter **(a)** and summer **(b)** of 2008. The drought year deformation patterns are shown in **(c)** for winter and **(d)** for the summer of 2014. The deformation patterns during the heavy precipitation year of 2017 are presented for winter **(e)** and summer **(f)**.


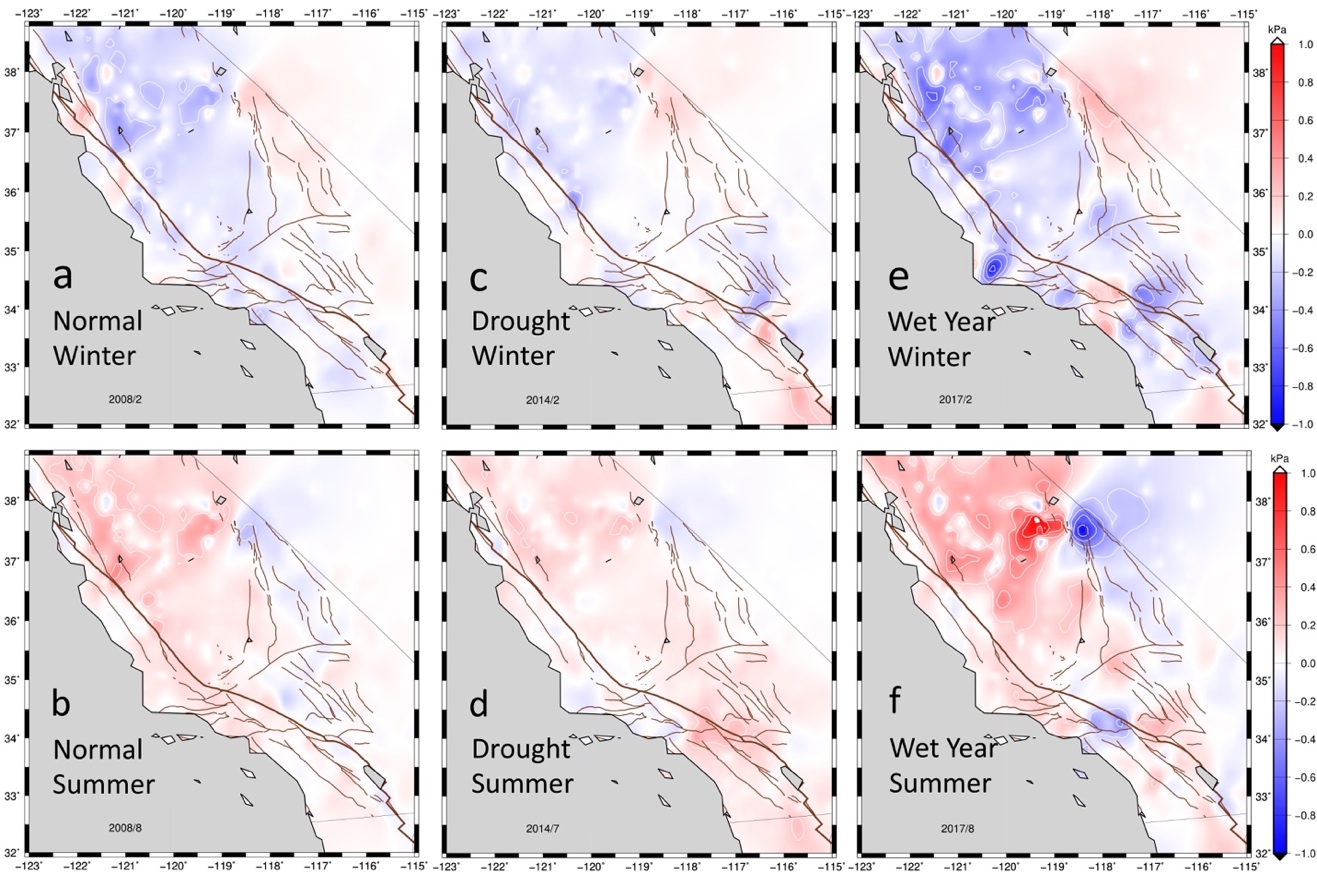


**Figure S3.** The associated Coulomb stress change with the long-wavelength transient horizontal strain obtained using cGPS data processed by CWU. The Coulomb stress changes are resolved on right lateral strike-slip faults (Kraner et al., 2018) for the normal winter **(a)** and summer **(b)** of 2008. The coulomb stress change for the drought year of 2014 are shown in **(c)** for the winter and **(d)** for the summer. The coulomb stress changes during the heavy precipitation year of 2017 are presented for winter **(e)** and summer **(f)**. The red is positive (fault-loaded) and the blue is negative (fault-released).

**Figure S4.** The number of cGPS datasets inverted to produce the long-wavelength transient strain solutions presented in Figure 3, Figure 4, Figure S1, Figure S2 as a function of time. Each of the orange asterisks is the number of available datasets for the corresponding time (MM-YYYY).


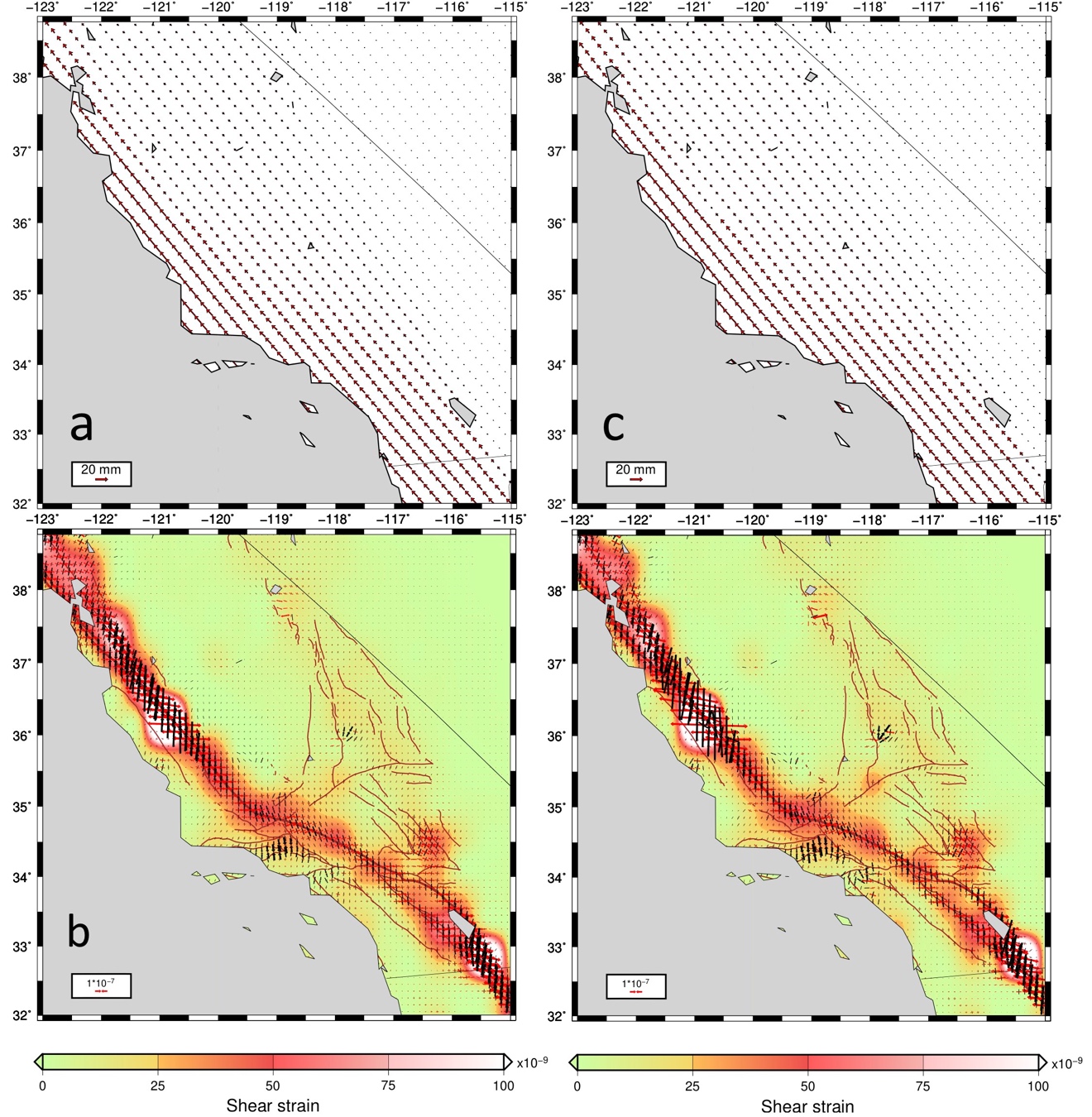


**Figure S5.** Continuous 4-month displacement field **(a)** and strain principal axes **(b)** of the 3-year average reference model. Contours are for the magnitude of shear strain associated with pure strike-slip strain. Another four-month cumulative continuous displacement field **(c)** and strain principal axes **(d)** derived from the steady-state strain rate tensor solution used by Kraner et al. (2018). The red vectors in **(a)** and **(c)** are the displacement fields relative to North American frame. The red and black axes in **(b)** and **(d)** indicate tensional and compressional principal axes of strain. The backgrounds show shear strain components.

**Figure S6.** Checkerboard test. **(a)** a 2º latitude x 4º longitude checkerboard pattern of synthetic dilatational strain and **(b)** recovered solutions. The magnitude of dilatational synthetic strain is ±22x10^-9^. **(c)** a 2º latitude x 1º longitude checkerboard pattern of synthetic dilatational strain of 33 and **(d)** recovered solutions. We add Gaussian errors (~0.1mm) to the synthetic horizontal displacements associated with the synthetic dilatation checkerboard patterns at the location of 678 NOTA stations. We invert the displacements for the recovered solutions **(b, d)**

**Figure S7**. Checkerboard test using a total field **(a)** that is the sum of the 2ºx4º and 2ºx1º fields presented in Figure S6**a**, S6**c**. We add Gaussian errors (~0.1mm) to the synthetic horizontal displacements associated with the total synthetic dilatation checkerboard pattern **(a)** at the location of 678 NOTA stations. We invert the displacements for the recovered solutions by applying **(b)** a higher damping (resulting in SEUW = 3) and **(c)** a lower damping (resulting in SEUW = 0). **(d)** Spatial correlations as a function of SEUW. The blue line shows the correlations between the synthetic total strain field in **(a)** and the recovered strain solutions. The red line shows the correlations between the synthetic long-wavelength strain field in Figure S6**a** and the recovered strain solutions. The black circles indicate the SEUW values for the solution in (**b)** (upper right) and **(c)** (lower right).

**Figure S8**. Time series at 10 GPS stations. Observed 4-month horizontal cGPS displacements (Yellow), corresponding long-wavelength models (light blue, on the left column) and shorter-wavelength model (Orange, on the right column). **(a)** P197; **(b)** P224; **(c)** P309; **(d)** P301; **(e)** P305; **(f)** P571; **(g)** P467; **(h)** FGST; **(i)** P612; and **(j)** BKAP. Each of the panels has time-series of the E-W component (top) and N-S component (bottom).


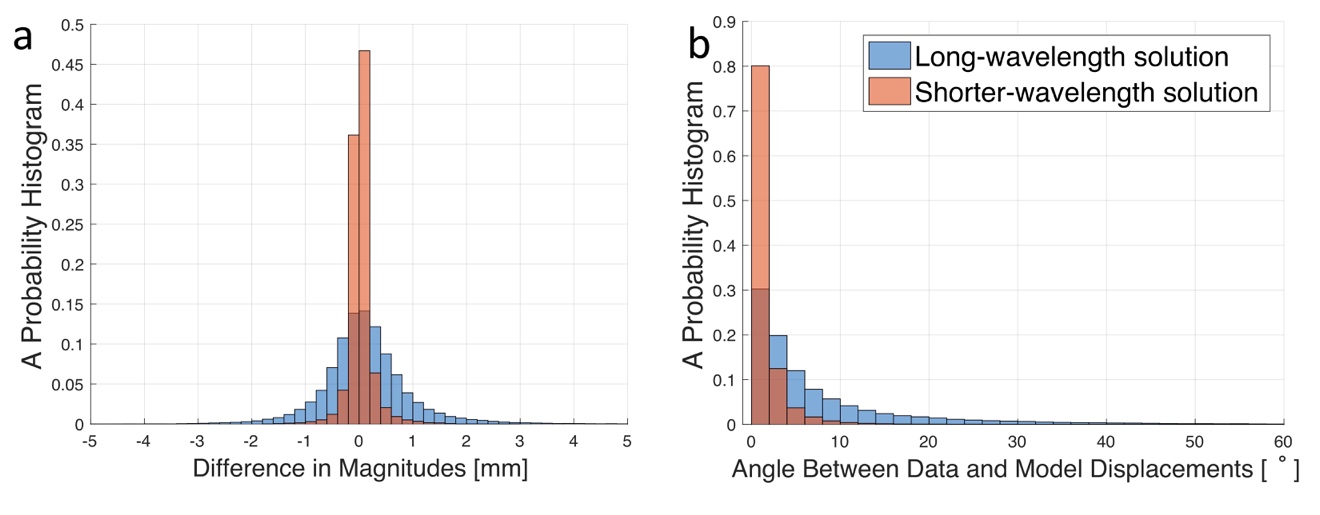


**Figure S9**. Probability Histograms for the misfits of magnitudes **(a)** and the angles **(b)** between the GPS displacements and the model displacements. The light blue bars are for the long-wavelength solutions and the orange bars are for the shorter-wavelength, higher-amplitude solutions.

**Figure S10.** Stacked model displacements relative to North America frame (NA12) obtained from cGPS with dilatational strains plotted in background for winter (a) averaged over 2/2008, 3/2008, 4/2008, 2/2009,3/2009,4/2009,2/2010,3/2010 and summer (b) averaged over 8/2007, 9/2007, 8/2008, 9/2008, 10/2008, 8/2009, 9/2009, 10/2009. We regard these stacked solutions as a representation of signal before M7.2 El Mayor-Cucapah earthquake signal. The stacked model displacements representing after-the-event deformation patterns are shown in (c) for winter averaged over February, March, and April of 2013, 2015, 2016, 2017, and in (d) for summer, averaged over August, September, and October of 2013, 2015, 2016, 2017, 2018. The positive dilatation is extensional. 2 sigma error ellipsoids are plotted. We stack both of the two hydrologic loading models before and after the El Mayor-Cucapah event to make comparisons (see Figures S11, S12). The focal mechanism in (c) and (d) is for the El Mayor-Cucapah Earthquake from Global Centroid-Moment-Tensor (GCMT) project (Dziewonski et al., 1981; Ekström et al., 2012).


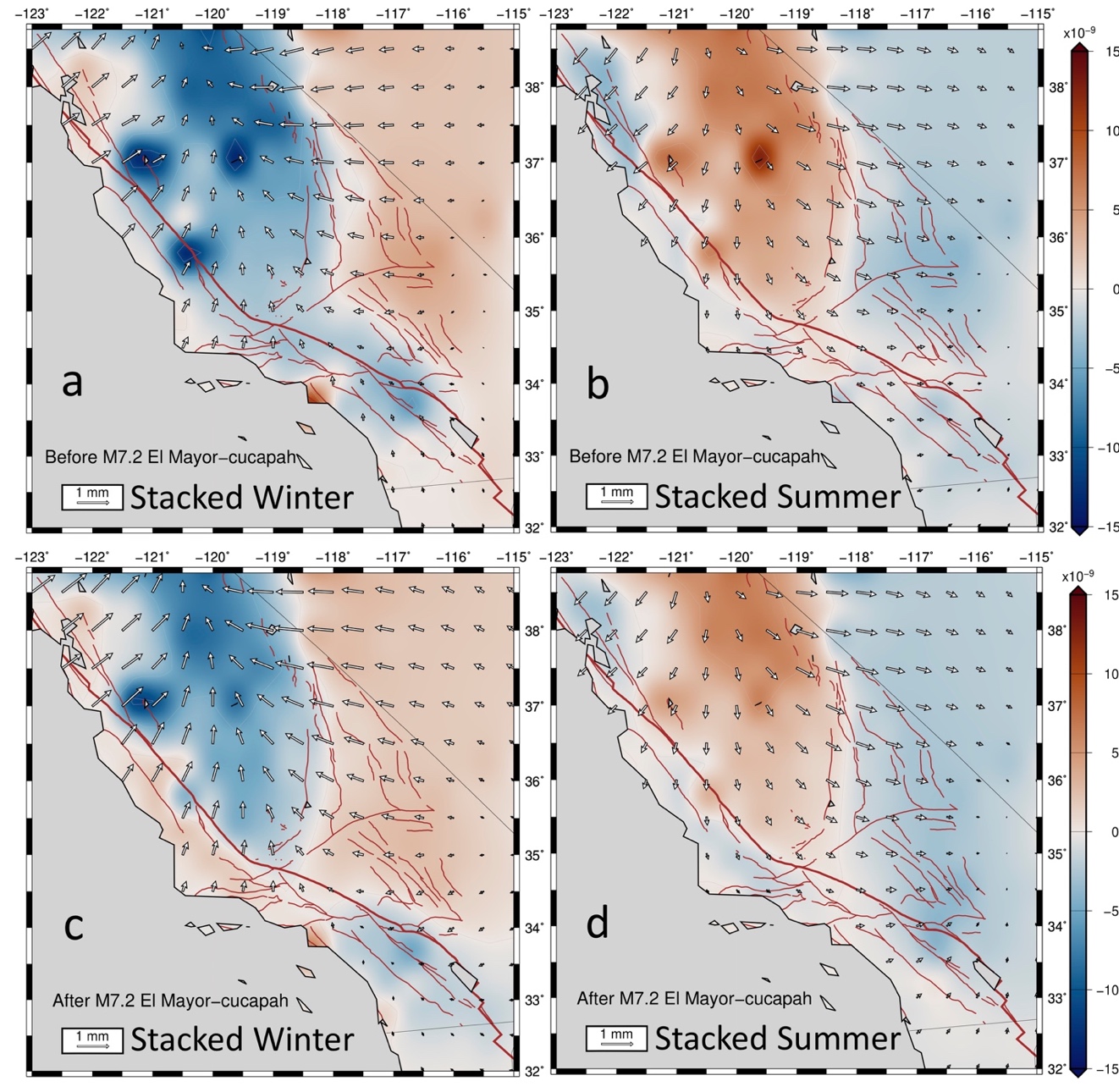


**Figure S11.** Stacked model displacements relative to “loading” frame obtained from surface water estimates (Argus et al., 2017) with dilatational strains plotted in background for winter (a) averaged over 2/2008, 3/2008, 4/2008, 2/2009,3/2009,4/2009,2/2010,3/2010 and summer (b) averaged over 8/2007, 9/2007, 8/2008, 9/2008, 10/2008, 8/2009, 9/2009, 10/2009. We regard this before M7.2 El Mayor-Cucapah earthquake signal. The stacked model displacements representing after-the-event deformation patterns are shown in (c) for winter averaged over February, March, and April of 2013, 2015, 2016, 2017 and in (d) for summer averaged over August, September, and October of 2013, 2015, 2016, 2017. The positive dilatation is extensional.


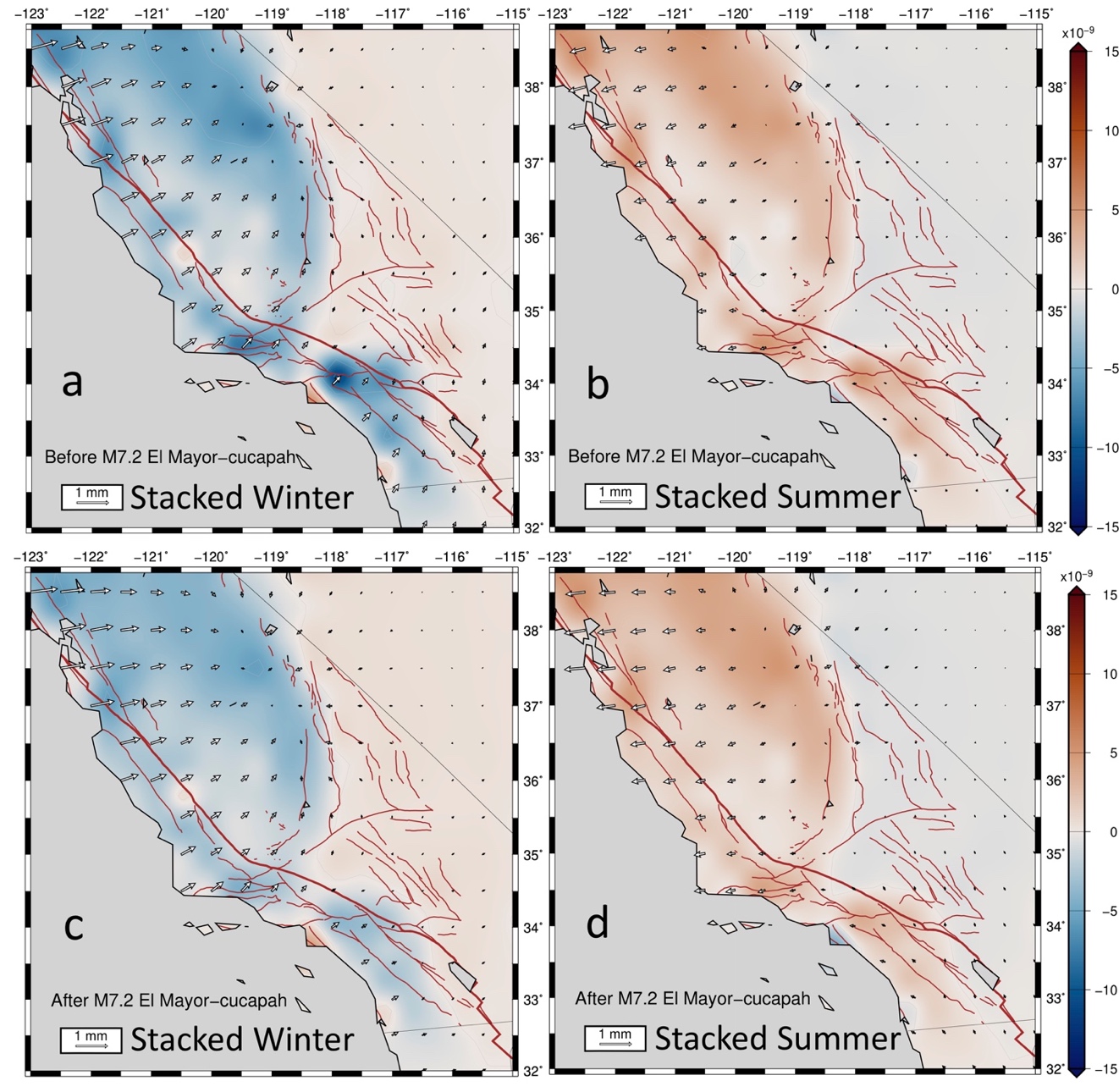


**Figure S12.** Stacked model displacements relative to “loading” frame obtained from UNAVCO hydrologic loading model (Puskas et al., 2017) with dilatational strains plotted in background for winter (a) averaged over 2/2008, 3/2008, 4/2008, 2/2009,3/2009,4/2009,2/2010,3/2010 and summer (b) averaged over 8/2007, 9/2007, 8/2008, 9/2008, 10/2008, 8/2009, 9/2009, 10/2009. We regard this before M7.2 El Mayor-Cucapah earthquake signal. The stacked model displacements representing after-the-event deformation patterns are shown in (c) for winter averaged over February, March, and April of 2013, 2015, 2016, 2017, and in (d) for summer averaged over August, September, and October of 2013, 2015, 2016, 2017. The positive dilatation is extensional.

**
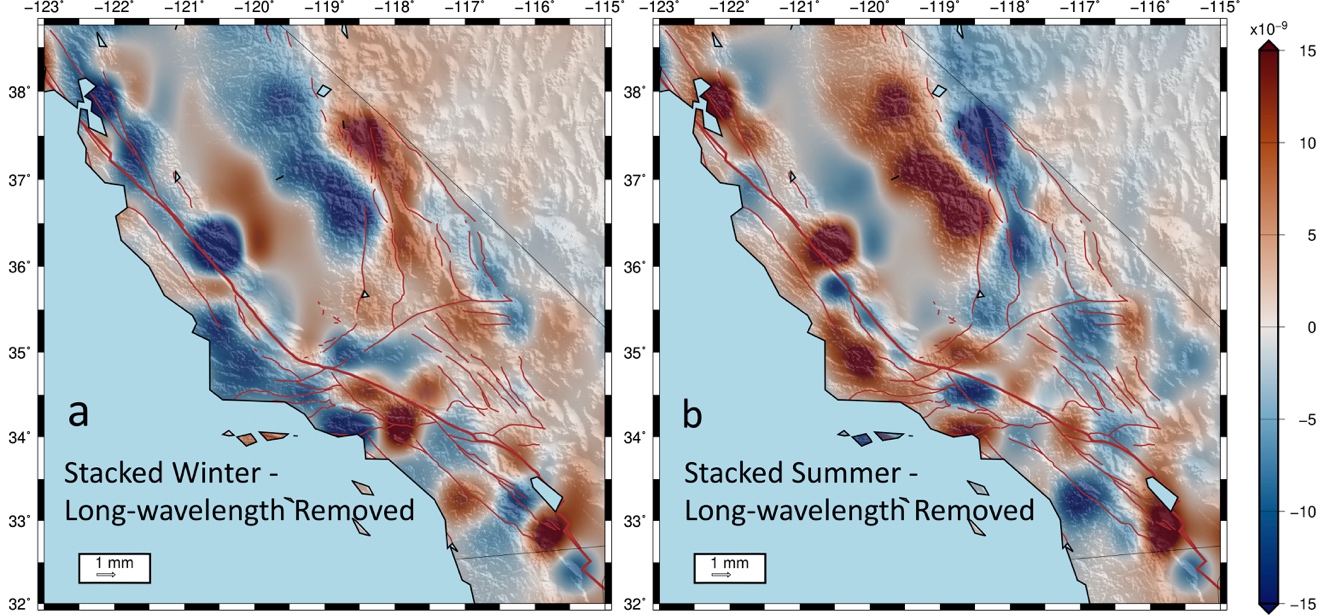
Figure S13**. Residual dilatational strain fields obtained after subtracting out the stacked long-wavelength solution from the stacked shorter-wavelength, higher-amplitude solution for  **(a)** winter and **(b)**summer. We plot the strain field with the shaded Digital Elevation Model (SRTM15+; Olson et al., 2014). The red is extensional and blue is contractional.

**
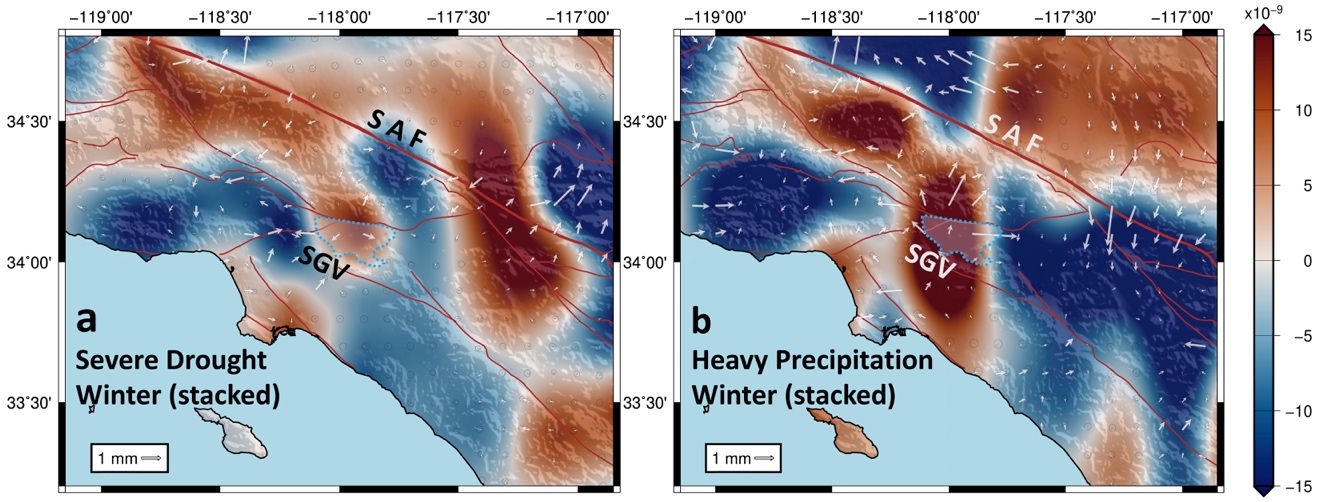
Figure S14**. Residual dilatational strain field between the shorter-wavelength, higher-amplitude solution and the long-wavelength solution in regions surrounding the San Gabriel Valley (SGV) for **(a)** average drought winter (over 1/2012, 2/2012, 3/2012, 1/2013, 2/2013, 3/2013, 1/2014, 2/2014, 3/2014, 1/2015, 2/2015 and 3/2015), and for **(b)** average heavy precipitation winter (over 1/2011, 2/2011, 3/2011, 4/2011, 2/2017, 3/2017, 4/2017, 3/2019, and 4/2019). The polygon made of blue dotted line indicates SGV. The red is extensional. The vectors are the residual displacements between the shorter-wavelength, higher-amplitude solution and the long-wavelength solution with 1 $\sigma$ error.

**
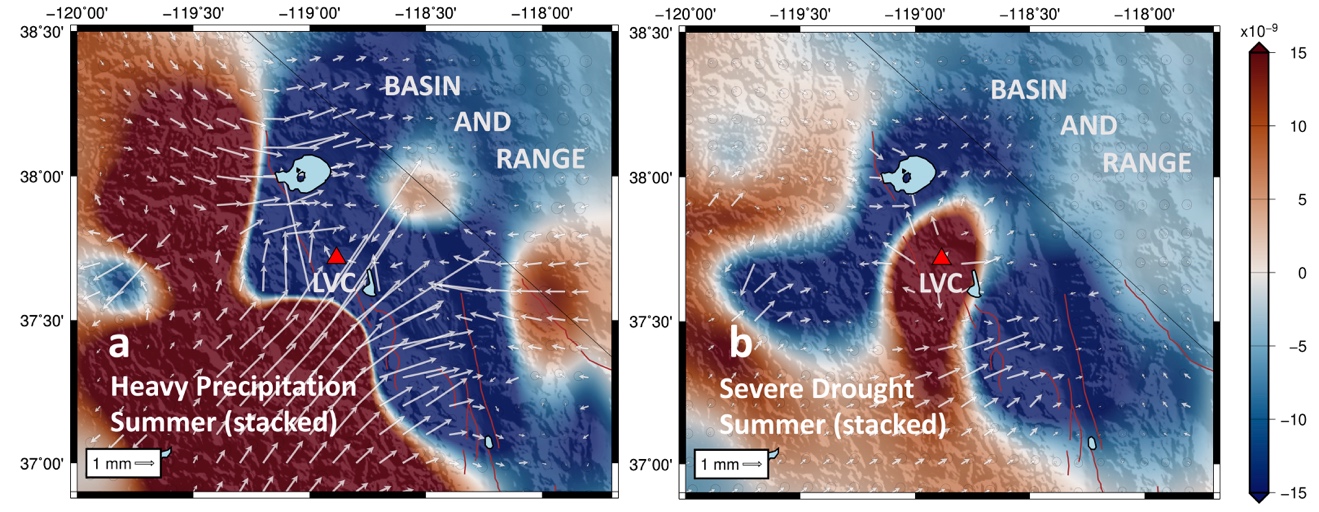
Figure S15**. Residual dilatational strain field between the shorter-wavelength, higher-amplitude solution and the long-wavelength solution in regions surrounding the Long Valley Caldera (LVC) for **(a)** average heavy precipitation summer (over 7/2011, 8/2011, 9/2011, 7/2017, 8/2017, and 9/2017), and for **(b)** average drought summer (over 7/2012, 8/2012, 9/2012, 7/2013, 8/2013, 9/2013, 7/2014, 8/2014, 9/2014, 7/2015, 8/2015, and 9/2015). The red triangle indicates LVC. The red is extensional. The vectors are the residual displacements between the shorter-wavelength, higher-amplitude solution and the long-wavelength solution with 1-$\sigma$ error.

**Figure S16.** Elastic Green’s function displacement response to loss of 1 meter water equivalent of a disk load (Wahr et al., 2013). The radius of the disk is 20 km. The black vertical dotted line indicates the edge of the disk load. The vertical displacement response is red solid line and the horizontal one is the blue solid line. We plot this figure using the ISSM-SESAW software (Adhikari et al., 2016). A tutorial to calculate the 3-D elastic response is available in the software package, which is available at: https://issm.jpl.nasa.gov. Note that the horizontal Green’s function response involves a change in sign of derivative (strain) at the edge of the disk load (vertical dotted line).


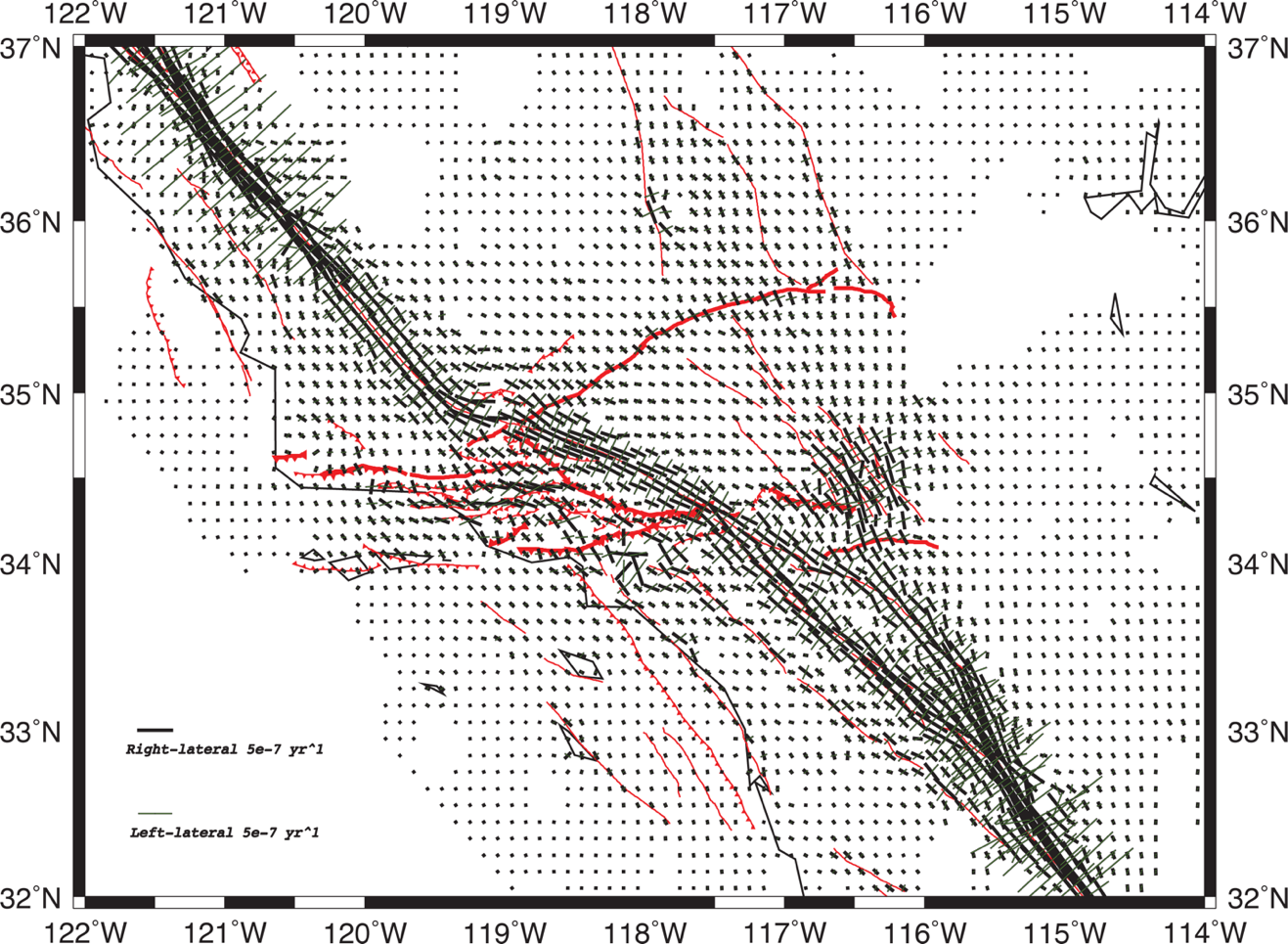


**Figure S17.** No-length-change directions obtained from the steady-state strain rate tensor solution defined from UCERF3 velocity field (Bold lines = Right-lateral shear directions, light green lines = left-lateral shear direction). Mapped major faults are shown in red (thick red lines = left-lateral; thin red lines = right-lateral; faults with teach have thrust component, and faults with dashes have a normal component). The no-length-change directions represent expected ‘fault orientations’ that we use to resolve Coulomb stress changes on (strike-slip fault orientation for vertical right-lateral fault). Note that the bold lines (right-lateral fault orientation) is generally close in orientation to the actual right lateral faults where mapped.

**Figure S18.** Integrated 1-month solutions over 4-month timespan. To make the comparisons with the 4-month solution in Figure 3 we plot the integrated 1-month solution for normal winter (a), normal summer (b); drought winter (c), drought summer (d); wet year winter (e) and wet year summer (f).


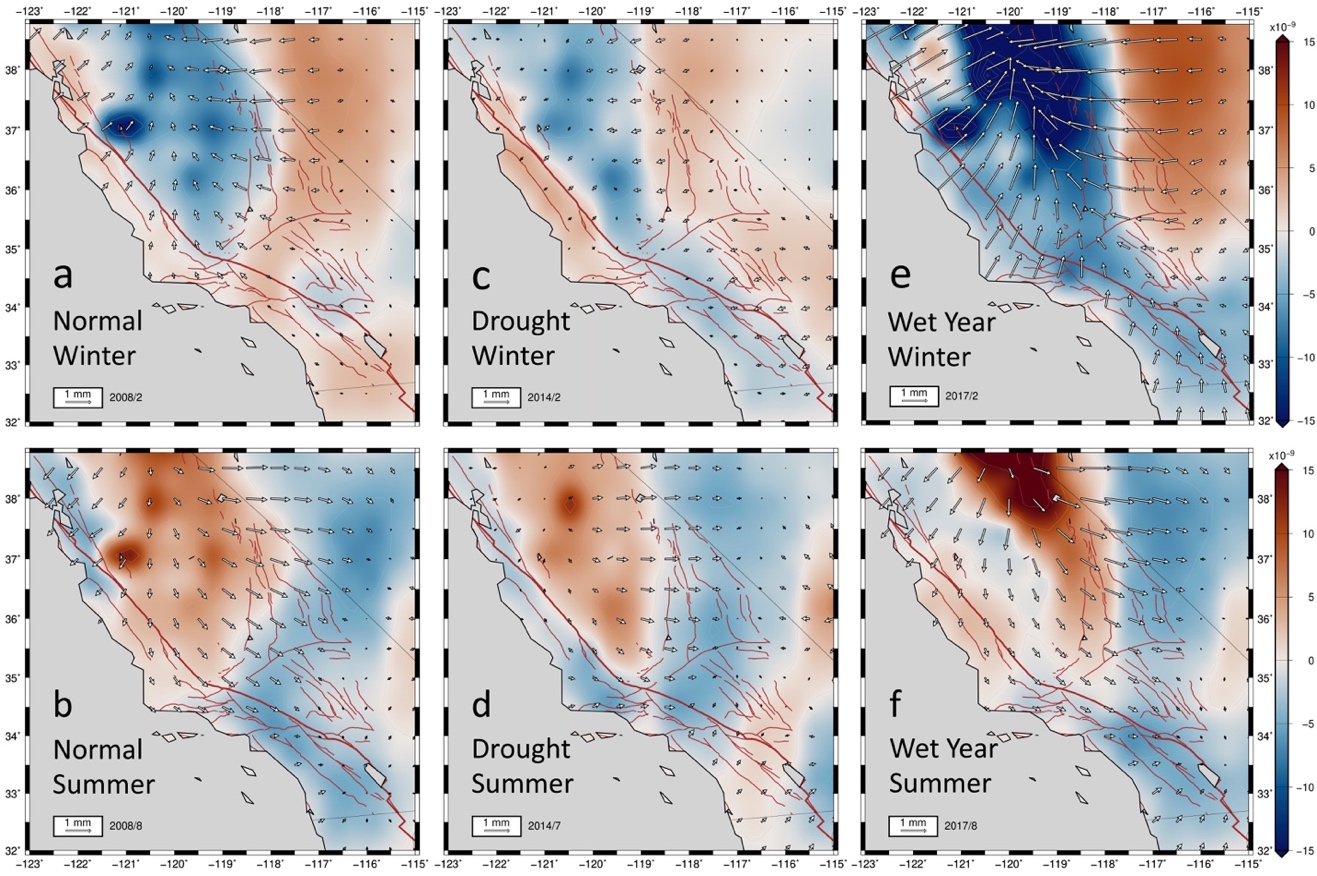


**Figure S19.** Model displacements relative to “loading” frame obtained from surface water equivalent estimates (Argus et al., 2017), with dilatational strains plotted in background for the normal winter **(a)** and summer **(b)** of 2008. The drought patterns are presented in **(c)** for the winter and **(d)** for the summer of 2014. Note the weak winter **(c)** and summer patterns **(d)** in comparison with the relatively larger signals for the normal winter **(a)** and summer **(b)** of 2008. The deformation patterns and motions during the heavy precipitation year of 2017 are shown in **(e)** for winter and **(f)** for summer. The positive dilatation (red) is extensional. Note that for these solutions the horizontal Green’s function responses (displacements) to the surface water loads are resolved on 0.1º intervals of latitude and longitude, instead of resolving on the location of the NOTA stations (compare with results presented in Figure 6).
